# All-visible-light-responsive porous aromatic frameworks manipulate CO_2_ uptake by reversible bulk isomerization of azobenzene pendants

**DOI:** 10.1073/pnas.2520024123

**Published:** 2026-01-30

**Authors:** Jinyu Sheng, Jacopo Perego, Silvia Bracco, Piotr Cieciórski, Wojciech Danowski, Angiolina Comotti, Ben L. Feringa

**Affiliations:** ^a^Stratingh Institute for Chemistry, Faculty of Science and Engineering, University of Groningen, Groningen 9747 AG, Netherlands; ^b^Department of Materials Science, University of Milano-Bicocca, Milan 20125, Italy; ^c^Faculty of Chemistry, University of Warsaw, Warsaw 02-093, Poland

**Keywords:** solid-state bulk photoswitching, CO_2_ uptake, porous materials, photoswitch, azobenzene

## Abstract

Embedding light-responsive small molecules in a porous solid is a promising strategy to achieve dynamic control over material properties. Powering these systems with low-energy photons is essential for their future applications, since visible light, compared to UV light, is less damaging and offers more selective isomerization with higher penetration depth. However, the construction of visible light-responsive porous materials remains a significant challenge. Here, we report the construction of a series of visible-light-responsive porous aromatic switchable framework materials grafted with *o*-fluoroazobenzene pendants (**Azo-PSFs**). The materials exhibit high microporosity and reversible photoswitching upon irradiation with visible light. The highly robust materials can be cycled between two distinct states multiple times without showing any photo fatigue or decomposition. Remarkably, solid-state NMR revealed that the azobenzene moiety undergoes reversible bulk isomerization in the framework. The isomerization of azobenzene within the framework is associated with substantial changes in adsorption capacity and CO_2_ uptake-release by the material. This work presents the example of visible-light-triggered bulk isomerization in an azobenzene-based porous material, providing a benchmark characterization of photoresponsive systems and paving the way for the future advancements in light-driven materials.

## Significance

Despite the clear advantages of low-energy photon activation, the development of porous materials that respond to visible light remains a significant challenge. Here, we report switchable porous aromatic frameworks (PAFs) appended with *o*-fluoroazobenzene that undergo reversible isomerization under alternating irradiation with distinct wavelengths of visible light. Solid-state NMR confirms that isomerization occurs throughout the bulk of the framework. This light-driven switching leads to marked changes in gas adsorption properties, including CO_2_ uptake. Notably, the relative modulation of CO_2_ uptake correlates with the content of the photoswitch. By demonstrating the example of visible-light-driven bulk isomerization in a porous solid, this work sets a benchmark for designing robust, light-responsive materials offering a pathway toward sustainable, visible-light-controlled gas storage and separation technologies.

The incorporation of molecular photoswitches into porous scaffolds represents one of the most promising strategies for constructing responsive materials with dynamically controllable properties. These systems combine the intrinsic porosity and high surface area of the scaffold with the on-demand responsiveness of the integrated photoactive molecules (1–5). Additionally, light provides exceptional spatiotemporal resolution, enabling precise, noninvasive control over the material properties. This unique combination of characteristics renders light-responsive porous materials particularly appealing for applications ranging from controlled gas uptake and release, guest diffusion to cargo delivery and molecular sieving (6–13). The seminal studies in recent years on the incorporation of photoswitches (14, 15) such as azobenzenes, spiropyrans, dithienylethenes, overcrowded alkenes, hydrazones, and donor–acceptor Stenhouse adducts moieties into porous scaffolds have provided a plethora of light-responsive metal-organic (MOFs) (16–24), covalent-organic (COFs) (25–28), porous aromatic (PAFs) frameworks (29–33) and other porous solids (34, 35). However, most of these materials require harmful, high-energy UV light to trigger photoisomerization of the embedded molecules (36, 37). Besides inducing unwanted side reactions that compromise the stability of the system, short-wavelength light also suffers from significant scattering and nonselective absorption, which severely restricts its penetration depth and hinders the efficient manipulation of these systems, i.e., limited or partial activation of photoswitch units inside the solid matrix. In contrast, visible light offers more selective and noninvasive excitation along markedly reduced scattering, which in principle should facilitate selective isomerization in the bulk of a solid (38–41). Although some visible-light responsive MOFs and COFs have been recently developed, visible light–responsive PAFs remain elusive. Unlike MOFs and COFs, which are assembled using more labile metal–ligand and dynamic covalent bonds, respectively, PAFs are constructed from robust C─C bonds (42, 43). This feature endows PAFs with exceptional chemical and thermal stability, making them ideal for application in conditions where the structural integrity of MOFs and COFs might be compromised (44). More importantly, in rigid, closely packed frameworks, particularly in layered 2D COFs, photoisomerization is often inefficient due to geometric constraints associated with strong π-stacking between adjacent layers (45, 46). As a consequence of these limitations, the process is primarily confined to the near-surface region, leaving the bulk of the material largely unaffected. In contrast, highly porous and flexible PAF-type networks can enable even sterically demanding isomerizations of various photoswitches. Notably, overcrowded alkenes incorporated into PAFs undergo quantitative isomerization in the bulk of the material (30, 31, 47), whereas this process is largely quenched in 2D COFs (48). Consequently, the development of visible-light-responsive PAFs capable of on-demand property changes would greatly advance the control and functionality of light-responsive porous materials and, as such, represents a major challenge.

**Fig. 1.**
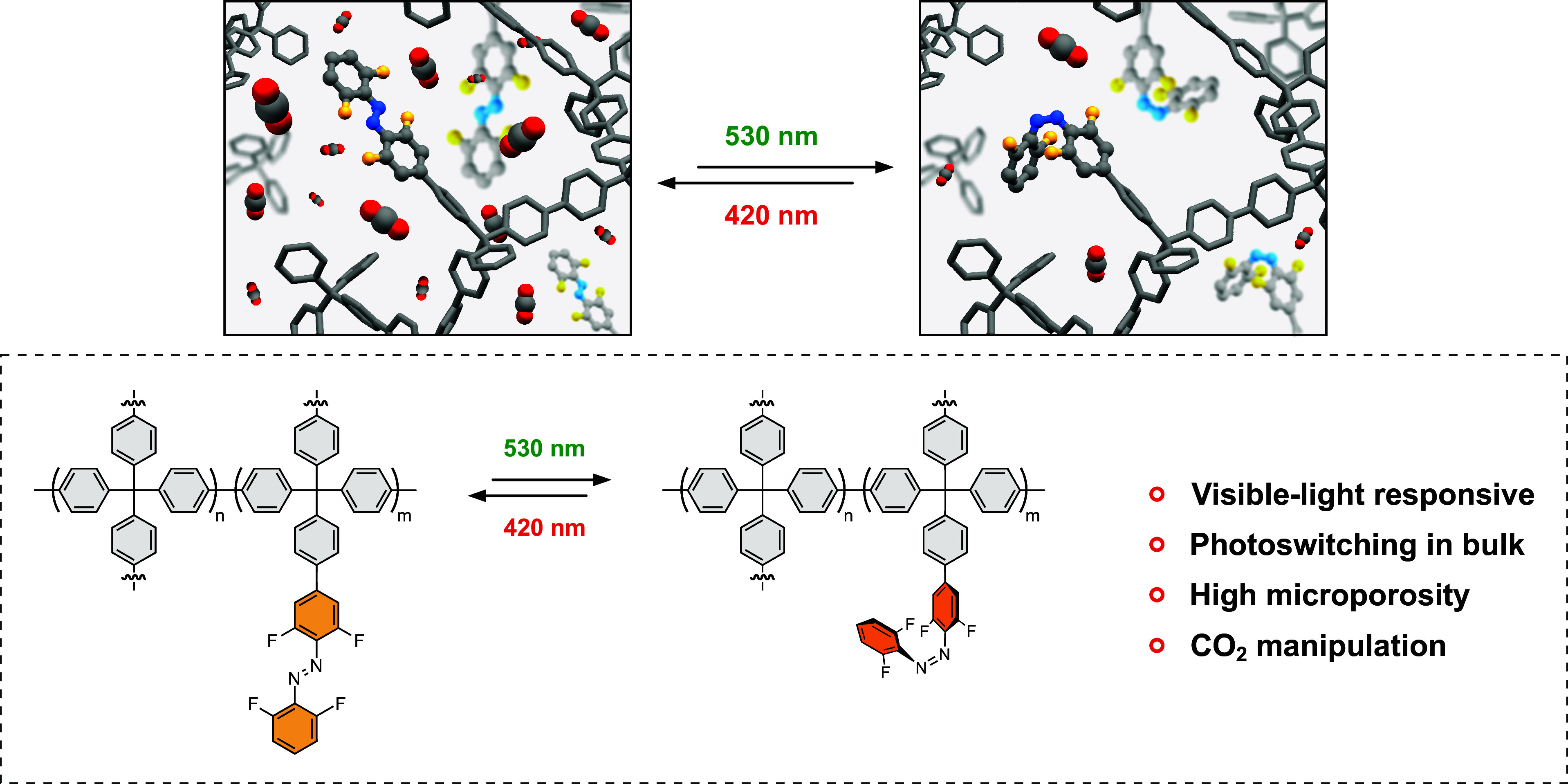
Schematic representation of visible-light-responsive porous aromatic framework (PAFs) materials enables CO_2_ uptake manipulation and the reversible bulk photoisomerization of grafted *o*-fluoroazobenzenes.

Azobenzenes are arguably the most prominent class of photoswitches, extensively studied for applications ranging from photopharmacology and catalysis to the development of responsive materials (49, 50). In particular, the recently developed *o*-fluorinated azobenzene variants are highly attractive due to their bidirectional visible-light-induced isomerization and exceptional thermal stability of the *Z*-form (51–54). Here, we demonstrate the design of a unique system comprising reversible, visible-light-triggered solid state photoisomerization of *o*-fluoroazobenzene incorporated into the porous aromatic scaffold denoted porous switchable framework (**Azo-PSF**) (Fig. 1). The materials were synthesized with Suzuki-Miyaura cross-coupling (SMC) using tetrafunctional porogenic units derived from tetraphenylmethane and a trifunctional photoswitch. Following this methodology, a series of materials with varying degrees of the photoswitch from 10 to 50% molar fraction were prepared. Though azobenzenes have been previously integrated into porous materials, direct evidence of visible-light-triggered bulk isomerization of azobenzene derivatives has never been provided. In the present work, the combination of diffuse-reflectance (DR) UV/Vis, diffuse-reflectance infrared Fourier transform (DRIFT), and solid-state ^19^F–^13^C and ^1^H–^13^C NMR (ssNMR) spectroscopies enabled the unambiguous quantification of bulk photoisomerization of *o*-fluoroazobenzene in the solid state. Remarkably, the photoswitching in the bulk enabled uptake modulation of various gases in the material bringing responsive porous solid materials to the next level of control.

## Results and Discussion

### Synthesis of Building Block E-1.

**Scheme 1.**
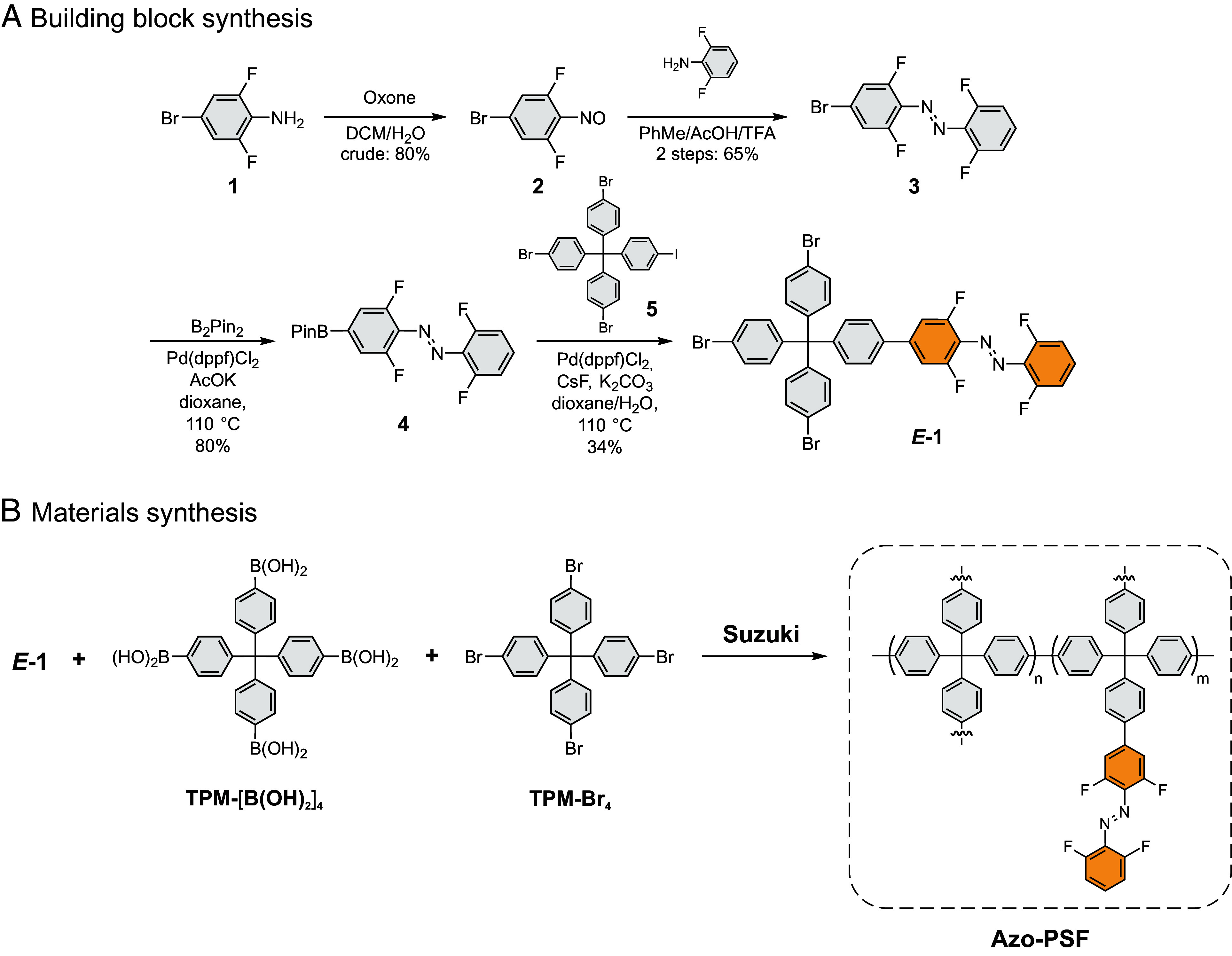
(*A*) Synthesis of building block ***E*****-1**. (*B*) Synthesis of **Azo-PSFs** by SMC reaction.

The visible-light-responsive **Azo-PSFs** were designed based on the *o*-fluorinated azobenzene photoswitch. The functional monomer was based on a tri-*p*-bromo-phenylmethane building block bearing, as a fourth side unit, a *p*-phenyl-tetra-*o*-fluoroazobenzene (denoted ***E*-1,** Scheme 1*A*). Unlike linear building blocks (35), this tri*-p*-brominated structure is expected to form a three-dimensional (3D) network with *o*-fluoroazobenzene units appended to the framework. The choice of a tribranched building block should ensure high pore capacity in the final material, thereby providing sufficient free volume for the unobstructed isomerization of the azobenzene units.

The synthesis of ***E*-1** started from commercially available aniline **1**, which was converted into the designed monomer in four simple steps with good overall yield (Scheme 1*A*). The azobenzene **3** was synthesized via a Baeyer–Mills reaction between nitroso compound **2**, and 2,6-difluoroaniline in 65% yield over two steps. Subsequent Miyaura borylation furnished the boron pinacol ester **4** in 80% yield. Finally, ***E*-1** was obtained in 34% yield in a SMC reaction between **4** and **5** (Scheme 1*A*). The identity of all the compounds was confirmed with ^1^H-, ^13^C-NMR, and HRMS (*SI Appendix*). Initial attempts to construct **Azo-PSFs** via Ullmann-type Yamamoto cross-coupling of ***E-1*** with the porogenic tetra-*p*-bromo-phenylmethane (**TPM-Br_4_**) failed, likely due to the strong reductive nature of Ni(0) species. As an alternative, Pd-catalyzed SMC reaction was adopted to construct the corresponding **Azo-PSFs** (Scheme 1*B*). Owing to the mild conditions, these cross-couplings are compatible with most moieties and are often employed for the direct synthesis of PAFs with diverse functionalities (43). Overall, three comonomers—porogenic **TPM-[B(OH)_2_]_4_**and **TPM-Br_4_** and functional ***E*-1,** were used to construct the **Azo-PSFs** materials (Scheme 1*B*). The monomers were used in stoichiometric ratios of bromides and boronic acids. Three distinct frameworks were synthesized, incorporating varying molar fractions of azobenzene groups and porogenic units—specifically with 1:10, 1:4, and 4:3 ratios of ***E*-1**:TPM (including both **TPM-[B(OH)_2_]_4_** and **TPM-Br**) in the frameworks denoted **^10^Azo-PSF**, **^20^Azo-PSF**, and **^50^Azo-PSF**, respectively (Table 1). These ratios were arbitrarily chosen to ensure sufficient free volume in the framework for unobstructed isomerization and to avoid intermolecular effects, e.g., stacking between adjacent photoswitches.

**Table 1.** Molar fractions of ***E*****-1** and two other building blocks used for the construction of **Azo-PSFs**

	*E*-1	TPM-[B(OH)_2_]_4_	TPM-Br_4_	*E*-1 fraction
^10^Azo-PSF	1.00	4.75	4.00	10%
^20^Azo-PSF	1.00	2.75	2.00	17%
^50^Azo-PSF	4.0	3.0	–	57%

### Characterization of Azo-PSFs.

**Fig. 2.**
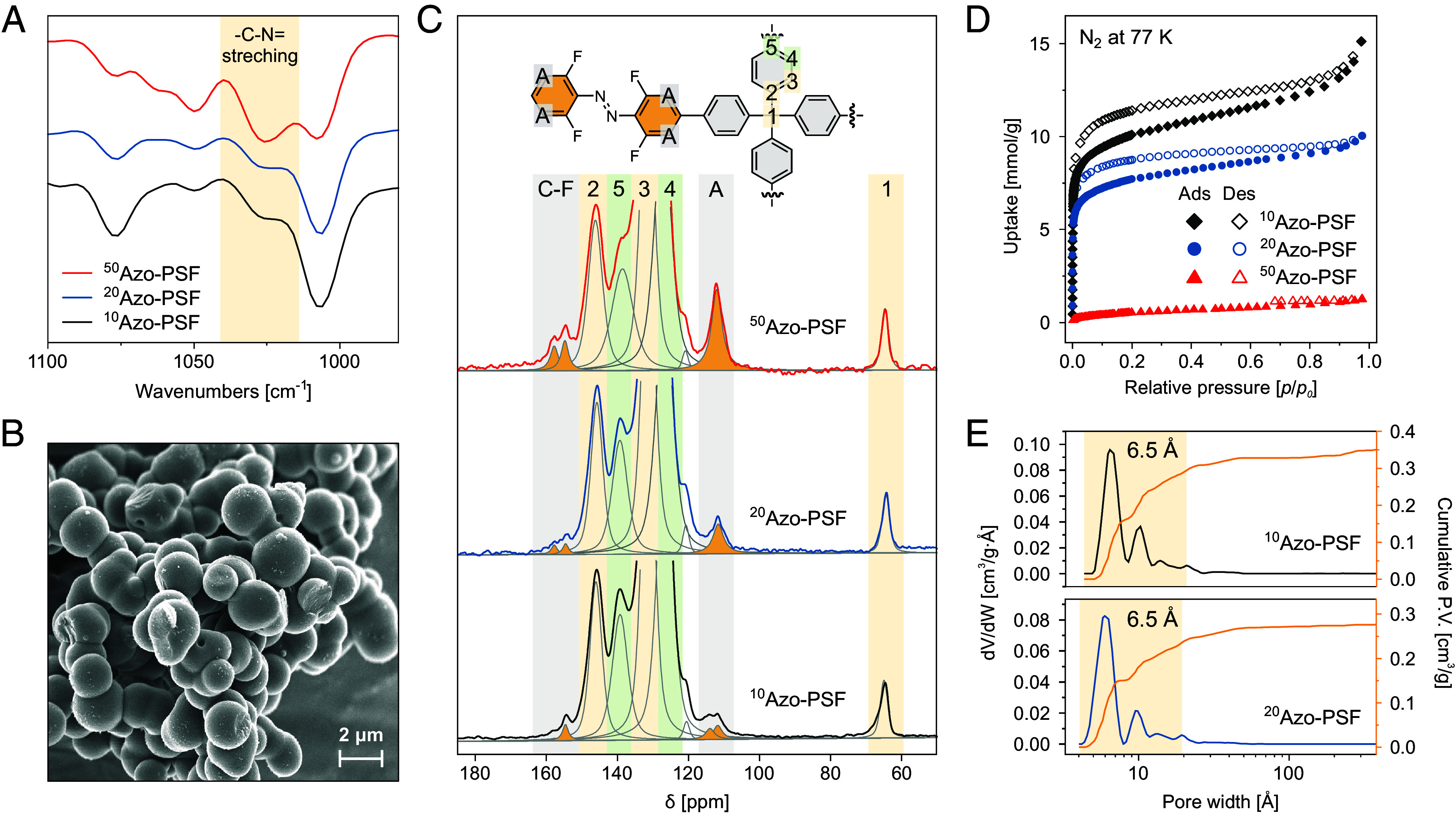
(*A*) Spectroscopic and textural characterization of **Azo-PSFs**. Selected IR region between 975 cm^−1^ and 1,100 cm^−1^, highlighting C–N bond stretching vibrations at 1,030 cm^−1^ for **^50^Azo-PSF** (orange line), **^20^Azo-PSF** (blue line), **^10^Azo-PSF** (black line), respectively. (*B*) SEM image of ^50^**Azo-PSF**. (*C*) ^1^H–^13^C CP MAS spectra of **Azo-PSF** materials collected with a contact time of 2 ms. (*D*) N_2_ adsorption/desorption isotherms of **Azo-PSFs** collected at 77 K. (*E*) Pore size distributions of **Azo-PSFs**. *Top*: differential (gray line) and cumulative (yellow line) pore size distribution of **^10^Azo-PSF**. *Bottom*: differential (blue line) and cumulative (yellow line) pore size distribution of **^20^Azo-PSF**. Micropore region (pore width <20 Å) is highlighted by the yellow shading. The pore size distributions were evaluated according to the HS-2D-NLDFT theory and carbon slit pore model. Note that the pore-size distribution for **^50^Azo-PSF** could not be determined due to negligible N_2_ uptake.

Successful materials fabrication as well as the presence of *o*-fluoroazobenzene in the final materials were confirmed by infrared spectroscopy (IR). Attenuated total reflection IR (ATR-IR) spectra showed a significant drop in the intensity of the bands centered at 3,600 cm^−1^ characteristic of O─H stretching in boronic acids. A small residual band was observed, indicating that subquantitative conversion of boronic acids, which is typical for PAFs constructed by Pd-catalyzed SMC reactions (*SI Appendix*, Fig. S5) (29). Likewise, a significant decrease in the intensity of the band characteristic of C–Br stretching centered at 1,050 cm^−1^ was observed. Both of these observations are consistent with the successful fabrication of the materials, largely consisting of the newly formed aromatic backbone with some residues of unreacted functional groups. The presence of the *o*-fluoroazobenzene in the materials was confirmed by the band centered at 1,030 cm^−1^ related to the C–N stretching, in agreement with the previously reported *o*-fluoroazobenzene incorporated into MOF materials (Fig. 2*A*) (18). Additionally, the increase of the intensity of the band centered at 1,030 cm^−1^ with the increasing ratio of *o*-fluoroazobenzene in the reaction mixture was observed, indicating a larger fraction of azobenzene incorporated into the materials. Elemental analysis of the materials (C, H, and N) showed that the fractions of the building blocks matched closely with those from the reaction feed (*SI Appendix*, Table S1). The residual percentage can be attributed to the presence of bromine atoms, which is typical for the PAF-type materials synthesized by SMC reactions.

Solid state ^1^H–^13^C cross polarization (CP) magic angle spinning (MAS) NMR spectra of the three samples enabled to identify the connectivity of the building blocks and unambiguously demonstrate the incorporation of the photoswitch in the framework (Fig. 2*C*). Specifically, the signal at approximately δ = 112 ppm—attributed to carbons bonded to hydrogen in the azobenzene moiety (denoted C_A_)- increased in intensity across the **^10^Azo-PSF**, **^20^Azo-PSF** and **^50^Azo-PSF** samples. The quantified content of the azobenzene corresponded to 10.8, 17.8, and 54.3%, respectively, in good agreement with the building block fractions in the polymerization feed. Additionally, the resonance at δ = 120.7 ppm indicated the presence of residual C-Br moieties, in agreement with ATR-IR data (*SI Appendix*, Fig. S5). Remarkably, the thermogravimetric analysis indicated exceptionally high thermal stability of the materials with no signs of decomposition up to 330 °C (*SI Appendix*, Fig. S6). Scanning electron microscopy (SEM) revealed that the **^10^Azo-PSF** and **^20^Azo-PSF** materials consist of isotropic spherical particles with diameters on the order of a few hundred nanometers (*SI Appendix*, Figs. S7 and S8), whereas the **^50^Azo-PSF** exhibits larger spherical particles, measuring up to a few micrometers (Fig. 2*B* and *SI Appendix*, Fig. S9). This morphology should ensure that even in larger particles, the bulk of the material can be effectively penetrated by light at wavelengths corresponding to the less intense absorption bands associated with n → π* excitation of azobenzene. Finally, powder X-ray diffraction indicated lack of any long-range order in the samples, as expected for the framework reticulated with an irreversible chemical reaction (*SI Appendix*, Fig. S10).

N_2_ sorption isotherms at 77 K revealed remarkably high surface areas for PAF-derived materials fabricated by SMC reaction, despite this method being less effective than Yamamoto polymerization at producing highly porous frameworks (*SI Appendix*, Table S2) (47). The Langmuir and Brunauer–Emmett–Teller (BET) surface areas of 934 m^2^ g^−1^ and 845 m^2^ g^−1^ for **^10^Azo-PSF** and 728 m^2^ g^−1^ and 648 m^2^ g^−1^ for **^20^Azo-PSF** were determined (Fig. 2*D*). The total pore volumes were estimated to be 0.49 and 0.32 cm^3^ g^−1^, respectively, with a consistent content of micropores of 0.30 and 0.23 cm^3^ g^−1^, respectively. The pore size distribution of the materials is centered at 0.65 nm (Fig. 2*E*), as calculated by Heterogeneous Surface 2-Dimensional Non-Local Density Functional Theory (HS-2D-NLDFT) and carbon slit pore model. The increase from 10 to 20% of monomeric unit ***E-*1** moderately reduces the porosity of the sample, while a drastic decrease of the N_2_ uptake at 77 K was observed for the **^50^Azo-PSF** due to the encumbrance of the azobenzene pendant groups protruding in the pores and the lower porogenic efficiency of the tripod building block in comparison to the tetrapodal TPM unit (*SI Appendix*, Table S2).

### Visible-Light-Triggered Photoswitching Behavior of *E*-1 in Solution.

**Fig. 3.**
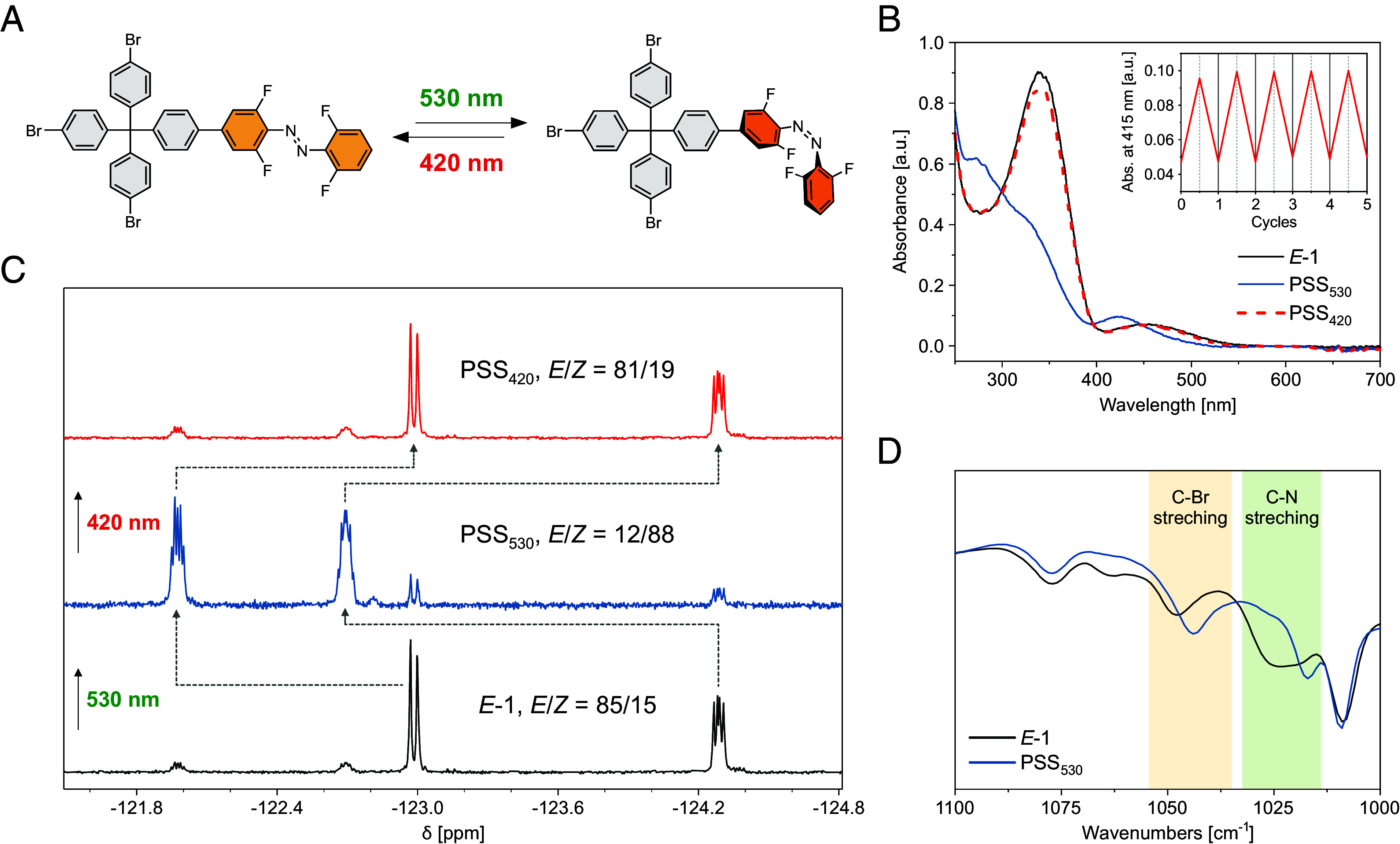
Isomerization of azobenzene switch in solution. (*A*) Photoisomerization of ***E*-1** to ***Z*-1** by 530 nm green light and back switching by 420 nm light (*B*) UV spectra from ***E*-1** (black) in CH_2_Cl_2_ to ***Z*-1** (blue) by 530 nm green light and backswitching by 420 nm light (dash red). *Inset*: Fatigue study of **1**. (*C*) ^19^F NMR spectra of ***E*-1** in CD_2_Cl_2_ (2 mM, RT) before irradiation (gray), after 530 nm green light irradiation (blue) and backswitching by 420 nm blue light (red). (*D*) Photoisomerization of ***E*-1** to ***Z*-1** by 530 nm light recorded by ATR-IR spectroscopy. A selected region is shown here to confirm the photoisomerization behavior of ***E*-1**.

The photochemical isomerization behavior of the monomer ***E*-1** in CH_2_Cl_2_ (Fig. 3*A*) was studied with ATR-IR, ^1^H-, ^19^F- NMR, and UV/Vis spectroscopies. Overall, the synthesized monomer showed high photoconversions upon reaching both photostationary states (PSS) and excellent reversibility of the isomerization, which is a hallmark of the *o*-fluorinated azobenzene scaffolds. In the UV/Vis spectra, irradiation of ***E*-1** at 530 nm led to a dramatic decrease of the intensity of the absorption bands centered at 330 nm and 475 nm, with the concomitant emergence of a new band centered at 425 nm characteristic of the formation of ***Z*-1** (Fig. 3*B*). The reversed photoisomerization from ***Z*-1** to ***E*-1** was observed upon irradiation of this mixture at 420 nm (Fig. 3*B*). During the whole process, clear isosbestic points were maintained at ca. 300, 397, and 448 nm, indicating a clean unimolecular photoisomerization (*SI Appendix*, Fig. S11). In addition, no signs of ***E*-1** photodecomposition were observed over five isomerization cycles upon alternating irradiations at 530 nm and 420 nm, further demonstrating the robustness of this photoswitch (Fig. 3*B*). ^19^F NMR spectra indicated that the as-synthesized azobenzene-appended monomer is a mixture of both *E*/*Z* isomers (85/15 molar ratio), likely due to the omnipresence of the ambient visible light (Fig. 3*C*, gray spectrum). Equilibration of this *E/Z* mixture at 110 °C in toluene for 16 h resulted in complete thermal isomerization of ***Z*-1** to ***E*-1**. Notably, the thermally generated ***E*-1** readily reverted to the as-synthesized *E*/*Z* mixture even under conditions of near-complete exclusion of ambient light, suggesting that this composition corresponds to the photostationary-state distribution of isomers under ambient white light (*SI Appendix*, Fig. S27). In contrast, a sample stored in total darkness exhibited no detectable isomerization, indicating that only the ***E*-1** isomer is present under thermal equilibrium (*SI Appendix*, Fig. S28). Irradiation at 530 nm led to a significant increase of the two downfield-shifted ^19^F resonances (chemical shifts from δ = −124.3 and −123.0 ppm to −122.7 and −122.0 ppm), in line with the formation of ***Z*-1**. Integration of these resonances allowed to determine the distribution of the *Z*/*E* isomers at the photostationary state (PSS_530_) of 88/12 (Fig. 3*C*, blue spectrum). Illumination of this mixture at 420 nm regenerated the ***E*-1** giving rise to a photostationary state mixture (PSS_420_) consisting of 81/19 *E*/*Z* (Fig. 3*C*, red spectrum). Finally, upon irradiation, the ATR-IR spectra exhibited significant shifts in the bands corresponding to C–Br and C–N stretching vibrations, shifting from approximately 1,050 cm^−1^ and 1,030 cm^−1^ to 1,045 cm^−1^ and 1,010 cm^−1^, respectively, consistent with the photochemical isomerization of azobenzene (Fig. 3*D*).

The thermal stability of the ***Z*-1** monomer was determined following the rate of the thermal ***Z*-1**→***E*-1** isomerization at five distinct temperatures (*SI Appendix*, Fig. S12). Extrapolation of the linear relationship to room temperature with Eyring plot provided the as calculated Gibbs free energy (Δ^‡^*G*° = 113.4 ± 0.2 kJ mol^−1^ K^−1^) of activation for the thermal isomerization (*SI Appendix*, Fig. S12) corresponded to the half-life of ***Z*-1** of *ca.* 100 d, indicating the high thermal stability of the ***Z*-1** in the experimental timeframe.

### Photoisomerization of Azo-PSF Materials in Solid State.

**Fig. 4.**
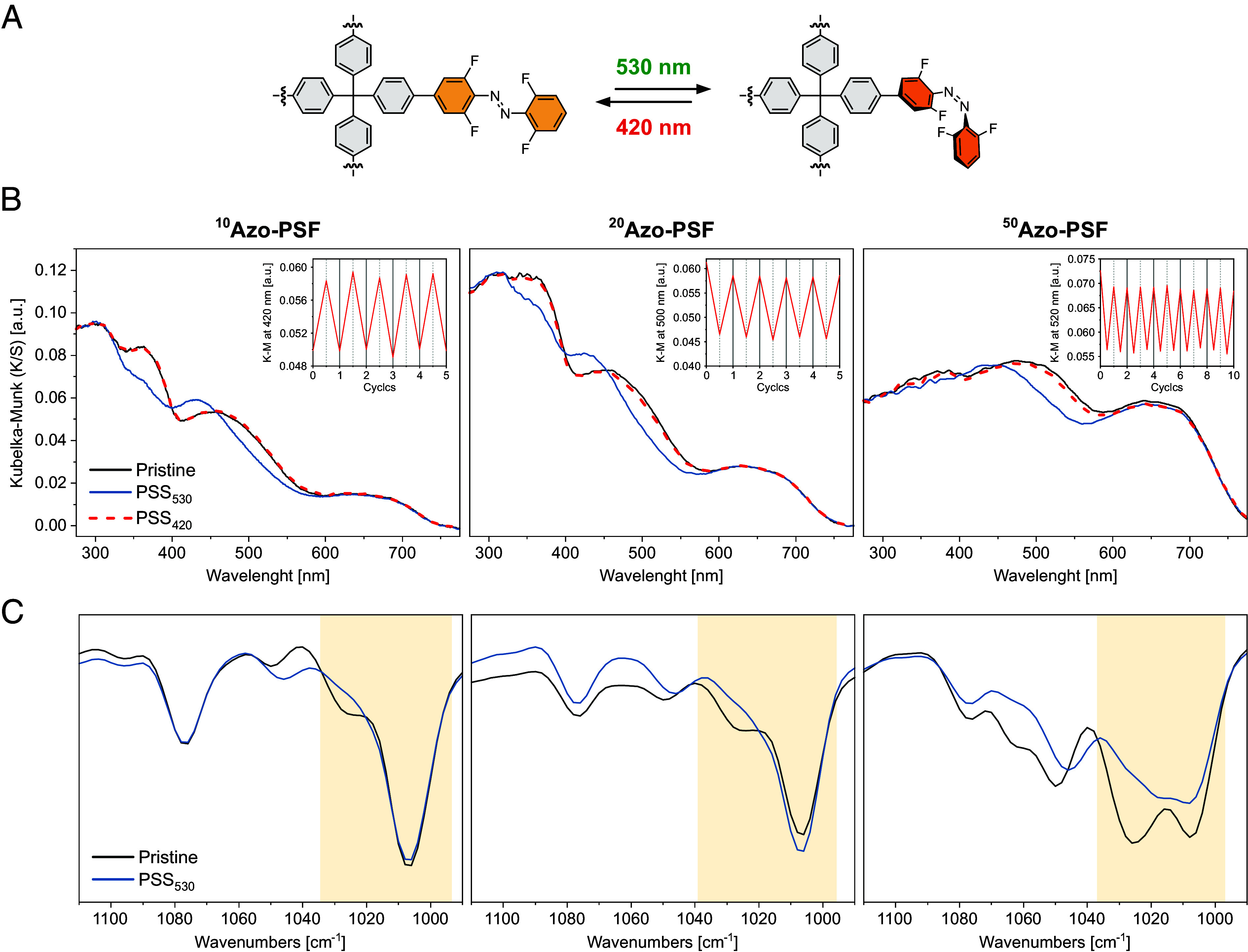
Isomerization of azobenzene switch in the solid state. (*A*) Schematic representation of the isomerization process in the bulk materials. (*B*) Photoswitching behavior of **Azo-PSFs** with different azobenzene ratios monitored by DR UV/Vis spectroscopy. Insets indicate the fatigue studies of materials by five or ten cycles of visible light manipulation (530/420 nm). (*C*) Photoswitching behavior of **Azo-PSFs** monitored with different azobenzene ratios by DRIFT spectroscopy at the region from 1,100 to 1,000 cm^−1^.

The photoisomerization of azobenzene in the porous solids (Fig. 4*A*) was studied by DR UV/Vis, DRIFT and solid-state NMR spectroscopies. In the DR UV/Vis spectra, a clear blue shift of the initial spectra was observed upon irradiation of the synthesized materials at 530 nm (**^10^Azo-PSF**, **^20^Azo-PSF,** and **^50^Azo-PSF**), indicating the photoisomerization of azobenzene from *E* to *Z* form in the solid state (Fig. 4*B*). Similar to the azobenzene in the solution, the backisomerization could be triggered upon irradiation of the materials at 420 nm. The near-perfect recovery of initial spectra confirmed the full regeneration of the *E* isomer of azobenzene. For all three solid frameworks, fully reversible photoisomerization was observed, indicating the amount of the azobenzene units incorporated into the porous network does not influence the photoswitching behavior, possibly owing to the sufficient degree of freedom in the material. In addition, irradiation at alternating wavelengths (530 and 420 nm) performed for several cycles showed no fatigue of the photoswitching behavior, thus highlighting photochemical robustness of these solid **Azo-PSFs** (Fig. 4 *B*, *Insets*).

DRIFT spectroscopy of the irradiated **Azo-PSFs** revealed similar spectral changes to those observed for ***E*-1** in solution, further confirming the *E*/*Z* photoisomerization of the azobenzene pendants in the solid. For all the frameworks, irradiation at 530 nm led to almost complete disappearance of the band associated with the C–N stretching centered at 1,030 cm^−1^. Noteworthily, the band characteristic of *Z*-azobenzene centered at 1,010 cm^−1^ was not observed, likely due to the overlap with an intense and broad band centered at 1,080 cm^−1^ originating from the aromatic ring “breathing” modes (Fig. 4*C* and *SI Appendix*, Figs. S13 and S14).

**Fig. 5.**
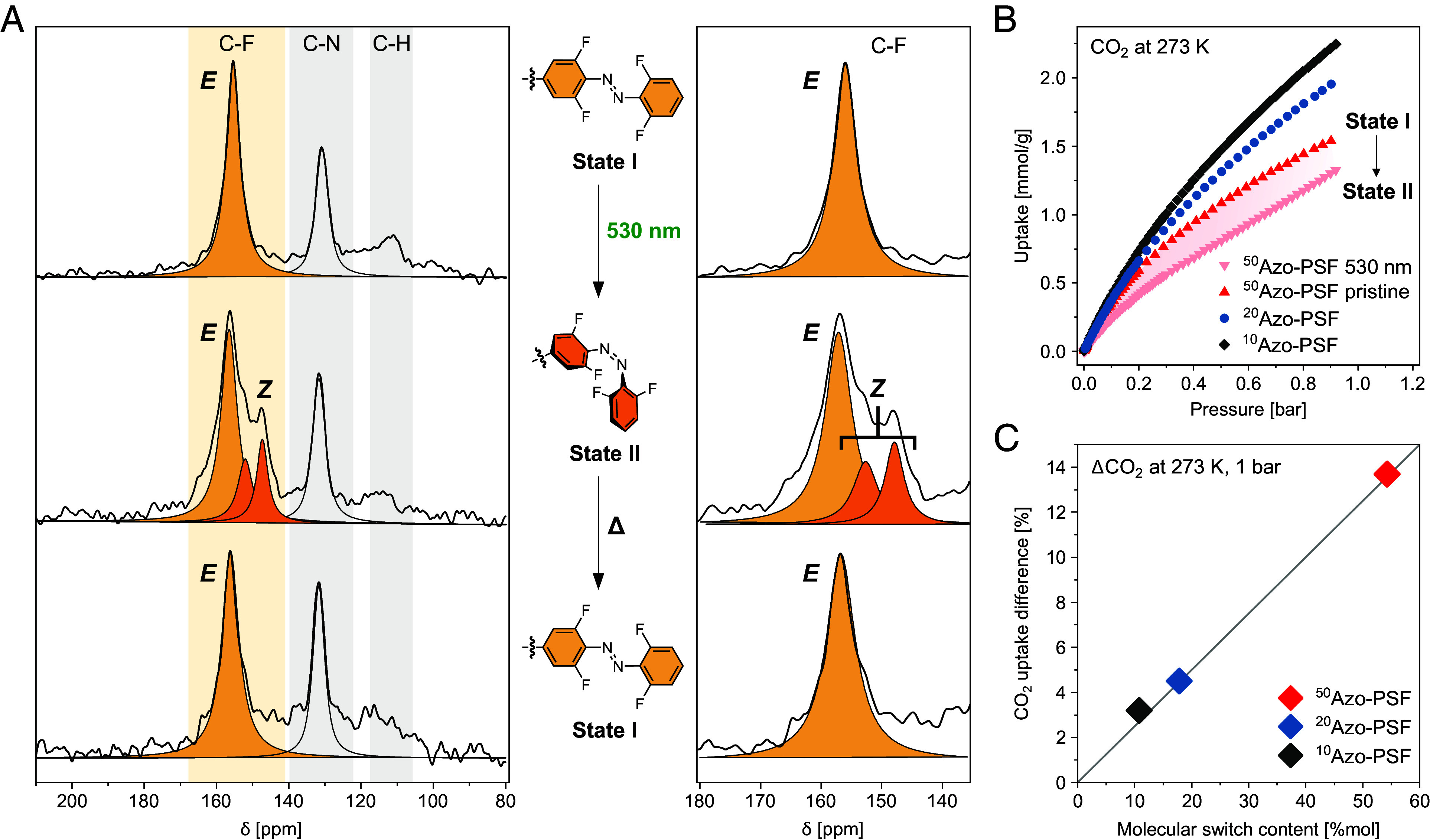
Bulk isomerization of azobenzene switch in the PSFs and their impact on controlling gas sorption modulations. (*A*) Photoswitching behavior of **^50^Azo-PSF** monitored by ^19^F-^13^C CP MAS NMR spectra (*Left*). Enlargement of the 135 to 180 ppm region to highlight *E-*to-*Z* isomerization (*Right*). (*B*) CO_2_ adsorption isotherms collected at 273 K of the pristine material **^10^Azo-PSF** (gray diamonds), **^20^Azo-PSF** (blue circles), and **^50^Azo-PSF** (red triangles, state I). CO_2_ adsorption isotherms at 273 K of **^50^Azo-PSF** after irradiation at 530 nm for 72 h (pink down-pointing triangles, state II). The pink shaded area highlights the modulation of CO_2_ uptake after irradiation. (*C*) CO_2_ uptake reduction after irradiation at 530 nm versus the molecular switch content of **Azo-PSFs**.

MAS NMR under cross polarization experiment from ^19^F to ^13^C enables the selective identification of carbons belonging to tetra-*o*-fluoroazobenzene pendant group. The resonance of carbon directly bound to fluorine atom (C–F) is sensitive to the photochemical isomerization from *E* to *Z* (Fig. 5*A* and *SI Appendix*, Figs. S15 and S16, see *SI Appendix* *f*or irradiation procedure of the bulk sample). In all three pristine samples a single signal at δ = 156 ppm was detected corresponding to the *E* isomer (state I) and, after irradiation at 530 nm, two upfield shifted resonances at δ = 152.2 and 147.5 ppm appeared, diagnostic of the formation of the *Z o*-fluoroazobenzene (state II) (32, 55). In line with the DR UV/Vis data, the extent of the *E*/*Z* isomerization in all **Azo-PSFs** was found to be similar, reaching from 36 to 38% of *Z* isomer with increasing the content of tetra-*o*-fluoroazobenzene moiety in the PSFs. The consistency of the photoisomerization behavior across all three materials suggests that the somewhat lower degree of photoisomerization in the solid framework compared with solution stems from interactions between the switchable pendants and the surrounding framework rather than from intermolecular interactions between adjacent azobenzene groups. After thermal treatment at 60 °C for 20 h and 100 °C for 4 h, the back isomerization to state I was proven by the recovery of the single signal characteristic of *E* isomer, demonstrating the reversibility of the process in the bulk of the materials. Furthermore, the *E*→*Z* photoisomerization of the azobenzene in the solid state was confirmed by ^19^F MAS NMR spectra, which show a downfield shift upon irradiation at 530 nm (*SI Appendix*, Fig. S17-S18), in agreement with ^19^F NMR in solution.

### Photomodulation of Porosity and Gas Uptake/Release of Azo-PSFs.

The modulation of gas uptake and porosity upon irradiation and thermal treatment of the **Azo-PSFs** was demonstrated by N_2_ and CO_2_ adsorption isotherms (Fig. 5*B* and *SI Appendix*, Figs. S19–S26 and Table S3, see *SI* *Appendix* for the irradiation procedure). The **Azo-PSFs** adsorb efficiently CO_2_ at 273 K with a maximum capacity at 0.9 bar of 2.2, 2.0, and 1.5 mmol g^-1^ for the **^10^Azo-PSF**, **^20^Azo-PSF**, and **^50^Azo-PSF**, respectively (Fig. 5*B*). After sample activation under mild conditions at room temperature, the adsorption properties were fully retained, as demonstrated by cyclic CO_2_ adsorption measurements (*SI Appendix*, Fig. S21). The sorption properties of **^10^Azo-PSF** have been investigated in detail. The isosteric heat of adsorption for CO_2_ molecules has been calculated as high as 26 kJ mol^-1^ at 0.1 mmol g^-1^, highlighting the good affinity between this guest and the framework (*SI Appendix*, Figs. S22 and S23). Moreover, the CO_2_ selectivity over N_2_ at 298 K was found to be 21, as calculated from Ideal Adsorbed Solution Theory, further highlighting the favorable interactions of the framework with CO_2_ guest molecules (*SI Appendix*, Figs. S22 and S24). Notably, the **^50^Azo-PSF** shows good CO_2_ uptake at 273 K, despite the low N_2_ uptake at 77 K, favored by the smaller kinetic diameter of CO_2_ and the more favorable diffusion kinetics at higher temperatures. The efficient uptake under mild conditions suggests the suitable pore size and the good affinity of the materials to CO_2_ molecules. The irradiation at 530 nm for 72 h led to a decrease of CO_2_ uptake (Fig. 5*B* and *SI Appendix*, Figs. S25 and S26). The effectiveness of this phenomenon appears to increase linearly on increasing *o*-fluoroazobenzene monomer unit content in the framework and reaches a remarkable 14% drop in CO_2_ uptake in the **^50^Azo-PSF** (Fig. 5*C*), representing one of the scarcely reported examples of photomodulation of CO_2_ uptake for a porous material under visible-light irradiation (*SI Appendix*, Table S4). Indeed, while the degree of photoisomerization in the solid state remains relatively constant for all the samples, the modulation of CO_2_ sorption increases with the higher density of *o*-fluoroazobenzene in **Azo-PSF** materials. The successful incorporation of a high content of appended photoswitch in the framework shows that its arrangement upon irradiation plays a key role in controlling the structure and size of the pores and in turn in modulating a macroscopic property at will such as the adsorption capacity of CO_2_ by an external stimulus.

### Conclusion.

In summary, the rational design of porous materials and monomeric unit enabled the fabrication of three all-visible-light-responsive, porous, switchable frameworks. The successful synthesis was achieved through a Suzuki-Miyaura cross-coupling reaction between a tripodal functional monomer and two tetrapodal porogenic units, yielding **Azo-PSFs** materials with intrinsically high microporosity. DR UV-Vis and DRIFT spectroscopies unequivocally confirmed the reversible photoswitching behavior of the *o*-fluoroazobenzene units within the solid frameworks. Additionally, solid-state NMR studies enabled to quantify the extent of photoisomerization occurring within the bulk material. Gas adsorption studies further demonstrated that the photoisomerization of the embedded switches drastically impacts the gas uptake capacity. Remarkably, the material with the highest azobenzene content and an almost negligible specific surface area exhibited the greatest extent of CO_2_ uptake photomodulation while maintaining a high overall CO_2_ adsorption capacity. This finding underscores the importance of rational design in functional porous frameworks for optimizing light-responsive gas storage and separation. These findings establish the concept of reversible, visible-light-triggered photoisomerization of a photochromic molecule within a scaffold derived from PAFs. Furthermore, these materials hold potential for use in visible-light-controlled CO_2_ uptake, release, and storage systems, contributing to environmental sustainability. Future research will focus on fine-tuning pore size via visible-light-induced photoisomerization and increasing the photoswitch loading to enhance selective gas separation and purification.

## Supplementary Material

Appendix 01 (PDF)

## Data Availability

All study data are included in the article and/or *SI Appendix*.
