# Supplementary material for: All-visible-light-responsive porous aromatic frameworks manipulate CO2 uptake by reversible bulk isomerization of azobenzene pendants
Source: Proc Natl Acad Sci U S A. 2026 Jan 30;123(5):e2520024123. doi: 10.1073/pnas.2520024123 (PMC12867705; doi:10.1073/pnas.2520024123)
Supplement: Supplementary file 1 — Appendix 01 (PDF) [file pnas.2520024123.sapp.pdf]

PNAS

Supporting Information for

All-Visible-Light-Responsive Porous Aromatic Frameworks Manipulate CO_2 Uptake by Reversible Bulk Isomerization of Azobenzene Pendants.

Jinyu Sheng,1,4,5,* Jacopo Perego,2,5 Silvia Bracco, 2 Piotr Cieciórski, 3 Wojciech Danowski,3,* Angiolina Comotti2,* and Ben L. Feringa4,*

1 Current address: College of Chemistry, Chemical Engineering and Materials Science, Soochow University, Suzhou, Jiangsu 215123, China

* E-mail: jysheng@suda.edu.cn

2 Department of Materials Science, University of Milano-Bicocca, Milan, Italy. Via R. Cozzi 55, Milan 20125, Italy.

* E-mail: angiolina.comotti@unimib.it

3 Faculty of Chemistry, University of Warsaw, Ludwika Pasteura 1, 02-093 Warsaw, Poland

*E-mail: w.danowski2@uw.edu.pl

4 Stratingh Institute for Chemistry, University of Groningen, Nijenborgh 3, 9747 AG Groningen, Netherlands.

* E-mail: b.l.feringa@rug.nl

5 These authors contributed equally.

This PDF file includes:

Supporting text

Figures S1 to S28

Tables S1 to S4

SI References

Contents

1. General Information 3
2. Synthesis... 5
___2.1. Switches synthesis. 5
___2.2. Material synthesis 9
3. Characterization of PSF materials 10
___3.1. Comparison of DRIFT IR spectra of PSFs 10
___3.2. Elemental analysis 10
___3.3. Thermogravimetry Analysis of ^{20}Azo-PSF 11
___3.4. SEM images of Azo-PSF materials 12
___3.5. Powder X-ray diffraction (PXRD) 15
___3.6 Adsorption properties 15
4. Isomerization studies 16
___4.1. Photoisomerization in solution 16
___4.2. Thermal isomerization studies of building block *E*-1. 17
___4.3. Reversible photoisomerization study of 50%-Azo-PSF. 18
___4.4. Solid State NMR spectra 19
___4.5. Gas adsorption modulation 22
___4.6. Photochemical isomerization of 1 under ambient light 25
5. References 28

1. General Information

All reagents were obtained from commercial sources and used as received without further purification. Dry solvents were obtained from an MBraun solvent purification system. Column chromatography was performed on a Reveleris X2 flash chromatography system. TLC: silica gel 60, Merck, 0.25 mm. High resolution mass spectrometry (ESI or APCI-MS) was performed on a LTQ Orbitrap XL spectrometer with ESI or APCI ionization. NMR spectra were recorded on Varian AMX400 (^{1}H: 400 MHz, ^{13}C: 101 MHz) and Varian Unity Plus (^{1}H: 500 MHz, ^{13}C: 126 MHz) spectrometers. Chemical shifts are quoted in parts per million (ppm) relative to the residual solvent signal (for $CDCl_3$ δ 7.26 for ^{1}H, δ 77.16 for ^{13}C and for CD_2Cl_2 δ 5.32 for ^{1}H, δ 53.84 for ^{13}C). For ^{1}H NMR spectroscopy, the splitting pattern of peaks is designated as follows: s (singlet), d (doublet), t (triplet), m (multiplet), br (broad), or dd (doublet of doublets). UV/Vis absorption spectra were measured on a Hewlett-Packard 8453 diode array spectrometer in a 1 cm quartz cuvette. DR UV/Vis spectra were measured Jasco V-570 UV/Vis NIR spectrophotometer equipped with Jasco ISN-470 integrating sphere. IR spectroscopy (ATR and DRIFT method) was performed on a PerkinElmer FT-IR Nexus spectrometer. All the irradiation experiments were performed using Thorlab LEDs (M365FP1, M420F2, M530F2). Solvents used for spectroscopic studies were of spectroscopic grade (UVASOL, Merck) or from a solvent purification system.

Elemental analysis (EA)

CHNS analysis was performed using an Elementar vario MICRO cube. The samples were treated overnight at 100°C under a high vacuum for at least 12 hours before CHNS measurements.

Solid State NMR spectroscopy

^{13}C and ^{19}F solid-state NMR experiments were carried out with a Bruker NEO 300 instrument operating at a static field of 7.04 T equipped a 4 mm double resonance MAS probe. ^{13}C{^{1}H} ramped-amplitude Cross Polarization (CP) experiments were performed at a spinning speed of 12.5 kHz using a recycle delay of 5 s and contact time of 2 ms. The 90° pulse for proton was 2.9 µs. Crystalline polyethylene was taken as an external reference at 32.8 ppm from TMS. ^{13}C{^{19}F} ramped-amplitude CP experiments were performed at a spinning speed of 12.5 kHz using a recycle delay of 8 s and contact time of 5 ms and 8 ms. The 90° pulse for fluorine was 2.5 µs. Quantitative solid-state ^{19}F MAS NMR spectra were performed at a spinning speed of 12.5 kHz using a recycle delay of 20 s. The ^{19}F chemical shift was referenced to sodium fluoride. Ex-situ sample irradiation: The samples were spread as a thin layer, ~ 0.5 mm thick, on a glass plate. Then, the bottom and the sides of the sample holder containing the Azo-PSFs were covered with aluminium foils, and the irradiation was performed from the top. The materials were agitated manually during the irradiation of the samples.

Thermogravimetry (TGA)

Thermogravimetric analyses were performed using a Mettler Toledo Star System 1 equipped with a gas controller GC10. Samples were outgassed overnight at 100°C under a high vacuum before the analysis to remove adsorbed species and inserted in a 70 µL alumina pan in air. TGA analysis performed under an oxidative atmosphere (dry air, flow rate = 50 mL/min) from 30°C to 1000°C highlighted the thermal stability of the samples.

Scanning electron microscopy (SEM) and sample preparation

Scanning electron microscopy (SEM) images were collected using a FEG Gemini 500 electron microscope (Zeiss). An accelerating voltage of 5 kV and a working distance of 4.2/4.3 mm were used for imaging. The sample was dried under vacuum and deposited on a conductive carbon tape. A thin layer of gold (10 nm) was sputtered onto the sample surface to enhance its electrical conductivity prior to SEM analysis.

Powder X-ray diffraction (PXRD)

Powder X-ray diffraction was collected on a Rigaku SmartLab diffractometer working in Bragg-Brentano geometry using Cu-Kα radiation, 40 kV, 30 mA over a range of 2θ (degree) = 5.0 - 60.0 with a step size of 0.02° and a scan speed of 1.0°/s.

Gas adsorption isotherms

N_2 adsorption isotherms at 77 K were collected up to 1 bar using a Micromeritics analyzer ASAP2020 HD. The samples were previously outgassed overnight at 100 °C under a high vacuum (10^{-3} mmHg) to remove the adsorbed species.

N_2 adsorption isotherms at 77 K were fitted using Langmuir and BET models. Differential and cumulative pore size distributions (PSD) were calculated according to the HS-2D NLDFT theory using the carbon slit pore model as implemented in the Microactive software (Micromeritics).

CO_2 adsorption isotherms at 273 K were collected up to 1 bar using a Micromeritics analyzer ASAP2020 HD. The temperature of the analysis was controlled using a Julabo F12-ED refrigerated/heating circulator.

For the photomodulation experiments, the activated samples were spread onto the flat bottom of the glass sample holder, forming a layer less than 0.5 mm thick. The sides of the sample holder were covered with aluminum foil, and the irradiation was performed from the bottom. The materials were stirred manually during the irradiation of the samples. Note that the sample was not removed from the sample holder to minimize errors in the adsorption isotherm related to sample weighing.

2. Synthesis.

2.1. Switches synthesis.

Building block **S2** was synthesized according to the reported literature procedure.[1]

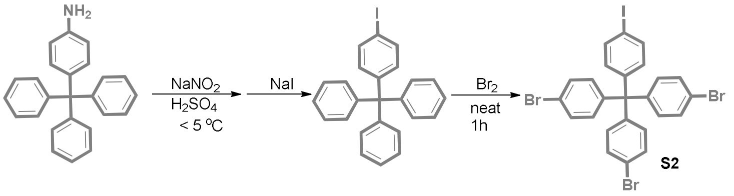

((4-iodophenyl)methanetriyl)tribenzene

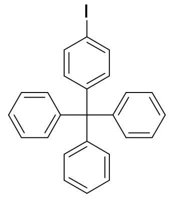

4-(Triphenylmethyl)-aniline (10 g, 29.8 mmol) was suspended in a mixture of conc. sulfuric acid and water with the ratio v/v=1:3 not exceeding a temperature of 50 °C. The suspension was cooled in ice bath and a solution of sodium nitrite (2.0 g, 29.8 mmol) in water (15 mL) was added dropwise to keep temperature lower than 5 °C. Then the yellow-colored suspension was added dropwise (25 min) into a solution of sodium iodide (4.5 g, 29.8 mmol) in water (15 mL). The brown-violet mixture was stirred for 1 h at room temperature and then additionally for 30 min at 100 °C. After the reaction mixture was cooled down to room temperature, 1M aqueous solution of sodium sulfite (approx. 200 mL) was added until the yellow color disappeared. After filtration of the suspension the filter cake was washed with water. The crude product was purified by column chromatography (SiO_2, DCM) to afford product as a yellow solid (9.5 g, 21.3 mmol, 71%). Analytical data in agreement with the literature.[1]

^{1}H NMR (400 MHz, $CDCl_3$) δ 7.66 – 7.60 (d, 2H), 7.34 – 7.22 (m, 15H), 7.07 – 7.01 (d, 2H).

4,4',4''-((4-iodophenyl)methanetriyl)tris(bromobenzene)

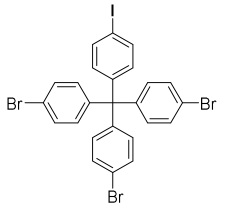

Iodo-4-(triphenylmethyl)-benzene (2.2 g, 4.9 mmol) was stirred in bromine (9 mL) for 1 h at room temperature. The mixture was cooled to 0 °C and cold ethanol (25 mL) was added. After dichloromethane (30 mL) and an aqueous solution of sodium thiosulfate (30 mL) were added the organic layer was separated and the aqueous solution was extracted several times with dichloromethane. The combined organic layers were concentrated under vacuum. The crude product was purified by column chromatography (SiO_2, pentane:DCM = 3:1 to 1:1) to afford product as a white solid (3.2 g, 4.7 mmol, 95%). Analytical data in agreement with the literature.[1]

^{1}H NMR (400 MHz, $CDCl_3$) δ 7.62 – 7.56 (d, 2H), 7.42 – 7.36 (d, 6H), 7.04 – 6.98 (d, 6H), 6.90 – 6.85 (d, 2H). **^{13}C NMR** (101 MHz, $CDCl_3$) δ 145.3, 144.6, 137.2, 132.8, 132.5, 131.2, 121.0, 92.6, 63.9.

Building block **TPM-[B(OH)$_2$]$_4$** was synthesized according to the literature reported proceduce.[2]

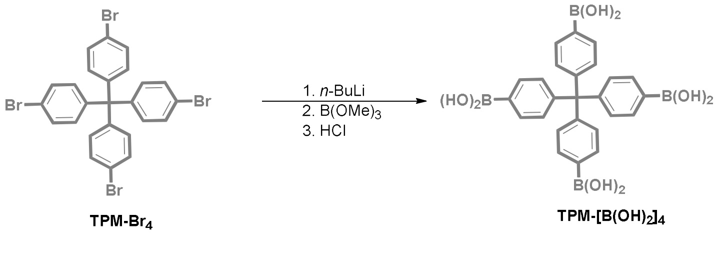

(Methanetetrayltetrakis(benzene-4,1-diyl))tetraboronic acid

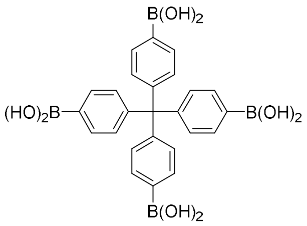

To the suspension of tetra(4-bromophenyl)methane (1.0 g, 1.6 mmol) in Et_2O (75 mL) was added a solution of n-BuLi in hexane (2.5 M, 3.8 mL, 18.9 mmol) via syringe at -78 °C. The resultant reaction mixture was heated to room temperature and stirred for 5 h. After cooling to -78 °C again, $B(OMe)_3$ (1.3 g, 12.6 mmol) was added to the reaction mixture, and the resultant mixture was allowed to warm to room temperature and stirred for 2 h. After removing the solvent by evaporation, to the resulting residue was added aqueous HCl solution (2 M, 10 mL) and the resultant mixture was filtrated. To the resulting solid was added aqueous NaOH solution (1 M, 30 mL) and the resultant mixture was filtrated. Then the pH value of the filtrate was adjusted to 5 by adding aqueous HCl solution (2 M). After filtration, the solid was washed with water and dried in vacuo to afford boronic acid as a white solid (0.7 g, 1.4 mmol, 88%). Analytical data in agreement with the literature.[2]

^{1}H NMR (300 MHz, DMSO-d_6) δ 8.00 (brs, 8H), 7.67 (d, *J* = 8.0 Hz, 8H), 7.13 (d, *J* = 8.0 Hz, 8H).

(E)-1-(2,6-difluoro-4-(4,4,5,5-tetramethyl-1,3,2-dioxaborolan-2-yl)phenyl)-2-(2,6-difluorophenyl)diazene.

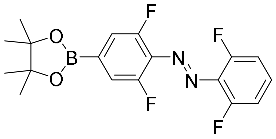

Compound **S1** was synthesized following the literature reported procedure.[3]

^{1}H NMR (300 MHz, $CDCl_3$)) δ 7.51 – 7.43 (m, 2H), 7.42 – 7.34 (m, 1H), 7.07 (t, *J* = 9.1 Hz, 2H), 1.36 (s, *J* = 1.0 Hz, 12H).

(*E*)-1-(3,5-difluoro-4'-(tris(4-bromophenyl)methyl)-[1,1'-biphenyl]-4-yl)-2-(2,6-difluorophenyl)diazene.

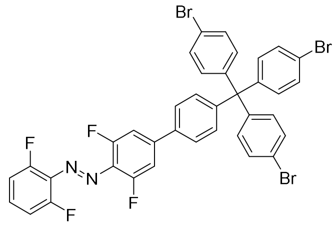

To a closed brown vial with a N_2 atmosphere, Pd(dppf)Cl_2 (73.1 mg, 0.1 mmol), CsF (760 mg, 2 mmol), K_2CO_3 (690 mg, 2 mmol), **S2** (1.36 g, 2 mmol) and **S1** (322mg, 1 mmol) were added. After addition of dioxane/H_2O (15 mL/6 mL), the mixture was purged with N_2 for 20 min before heated to 110 °C. The reaction mixture was heated at reflux (110 °C) for 24 h. The mixture was quenched with H_2O (50 mL) and the product was extracted with EtOAc (3*50 mL). The combined organic layers were washed with brine (50 mL), dried over Na_2SO_4 and concentrated under vacuum. The crude product was purified by column chromatography (SiO_2, pentane:DCM = 5:1 to 1:1) to afford **1** as red waxy solid (364 mg, 0.45 mmol, 45%). **Note:** The reaction mixture should be covered with Aluminum foil, and all work-up steps should avoid long-time room light exposure as otherwise the compound will isomerize (as observed by ^{1}H NMR).

^{1}H NMR (400 MHz, CD_2Cl_2) δ 7.59 – 7.56 (m, 2H), 7.47 – 7.41 (m, 7H), 7.37 – 7.29 (m, 4H), 7.14 – 7.09 (m, 8H). **^{13}C NMR** (101 MHz, CD_2Cl_2) δ 157.7, 157.7, 157.3, 157.3, 155.2, 155.1, 154.7, 154.7, 147.1, 145.2, 145.0, 144.9, 144.8, 144.7, 136.2, 136.2, 136.2, 132.9, 132.8, 131.8, 131.5, 131.5, 126.9, 121.0, 64.3. **^{19}F NMR** (282 MHz, $CDCl_3$) δ -119.84 (d, *J* = 10.7 Hz), -121.15 (dd, *J* = 9.3, 5.9 Hz). **HRMS** (ESI pos) calcd $C_{37}H_{32}Br_3F_4N_2$ $[M+H]^+$: 808.9243, found 808.9225.

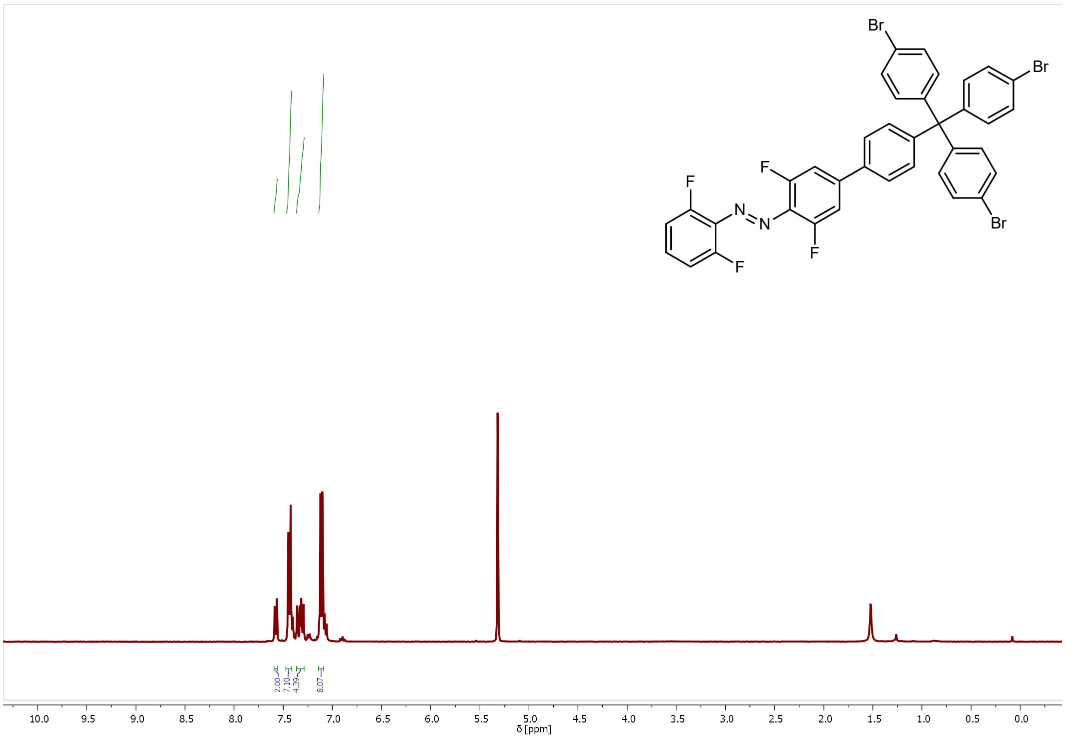

Fig. S1: ^{1}H-NMR spectrum of *E*-**1** measured in CD_2Cl_2 at 298 K.

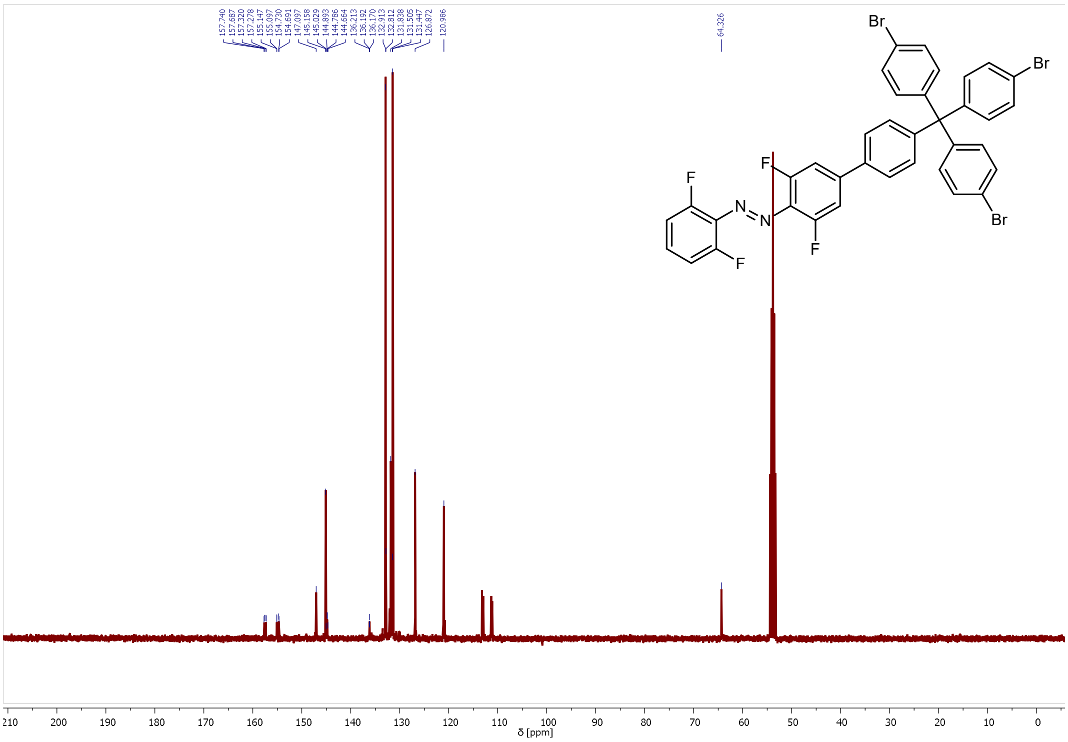

Fig. S2: ^{13}C-NMR spectrum of *E*-**1** measured in CD_2Cl_2 at 298 K.

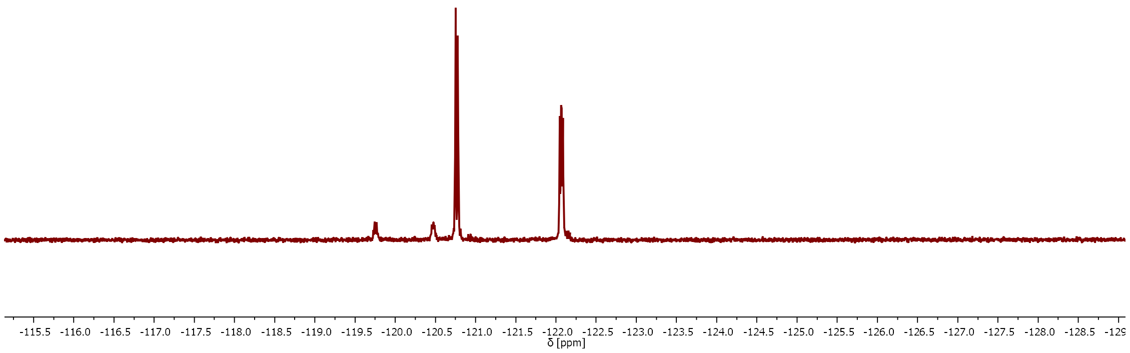

Fig. S3: ^{19}F-NMR spectrum of the *E*-**1** measured in CD_2Cl_2 at 298 K.

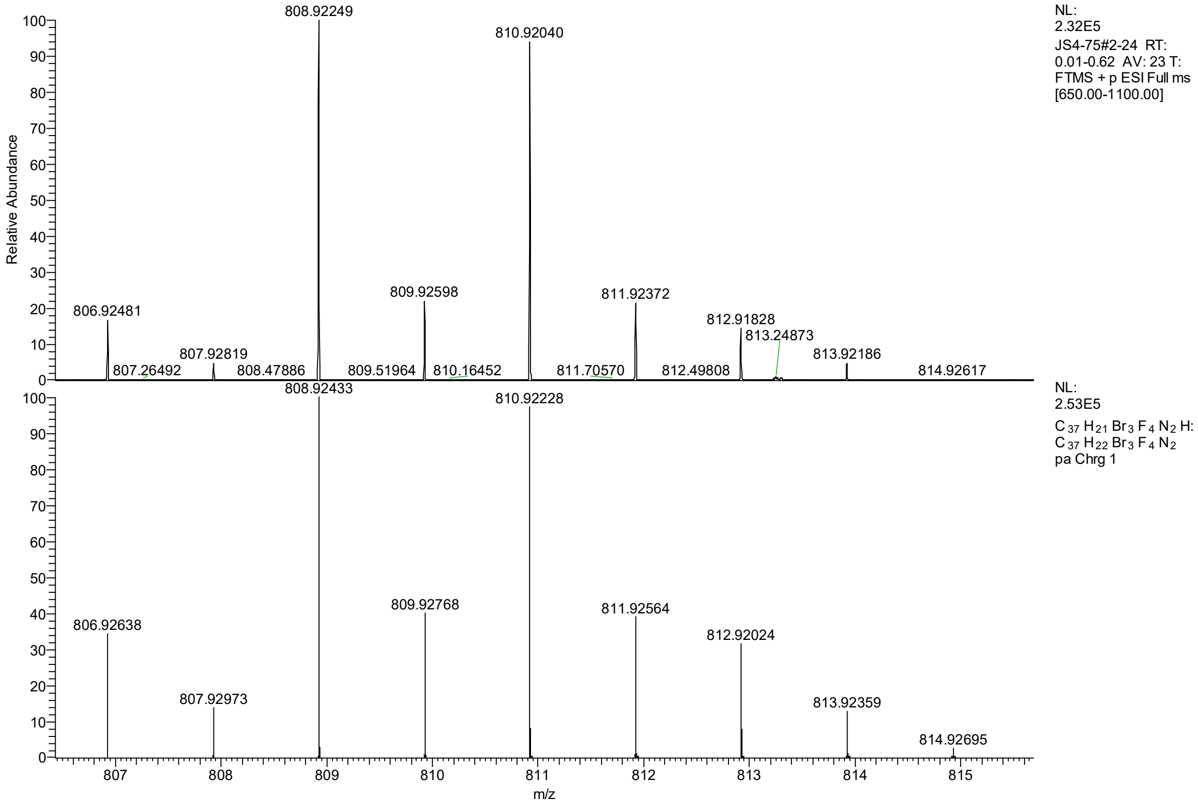

Fig. S4: HR-MS spectrum of *E*-**1** (top:measured, bottom: calc.).

2.2. Material synthesis

10%-Azo-PSF synthesis. To a closed brown vial with a N_2 atmosphere, $Pd(PPh_3)_4$ (4.2 mg, 0.004 mmol), K_2CO_3 (151.8 mg, 1.1 mmol), TPM-Br_4 (50.5 mg, 0.08 mmol), TPM-$[B(OH)_2]_4$ (47.1 mg, 0.095 mmol) and **1** (16.2 mg, 0.02 mmol) were added. After addition of THF/H_2O (5 mL/5 mL), the mixture was purged at N_2 for 20 min. The reaction mixture was heated at reflux (75 °C) for 24 h. The insoluble orange/brown polymer was washed with THF, H_2O, MeOH, DCM and THF (20 mL each), and dried at 80 °C under vacuum to afford the material quantitatively for further characterization.

20%-Azo-PSF synthesis. To a closed brown vial with a N_2 atmosphere, $Pd(PPh_3)_4$ (4.2 mg, 0.004 mmol), K_2CO_3 (151.8 mg, 1.1 mmol), TPM-Br_4 (50.5 mg, 0.08 mmol), TPM-$[B(OH)_2]_4$ (54.5 mg, 0.11 mmol) and **1** (32.4 mg, 0.04 mmol) were added. After addition of THF/H_2O (5 mL/5 mL), the mixture was purged at N_2 for 20 min. The reaction mixture was heated at reflux (75 °C) for 24 h. The insoluble orange/brown polymer was washed with THF, H_2O, MeOH, DCM and THF (20 mL each), and dried at 80 °C under vacuum to afford the material quantitatively for further characterization.

50%-Azo-PSF synthesis. To a closed brown vial with a N_2 atmosphere, $Pd(PPh_3)_4$ (3.0 mg, 0.0024 mmol), K_2CO_3 (82.3 mg, 0.6 mmol), TPM-$[B(OH)_2]_4$ (29.8 mg, 0.06 mmol) and **1** (64.6 mg, 0.08 mmol) were added. After addition of THF/H_2O (5 mL/5 mL), the mixture was purged at N_2 for 20 min. The reaction mixture was heated at reflux (75 °C) for 24 h. The insoluble orange/brown polymer was washed with THF, H_2O, MeOH, DCM and THF (20 mL each), and dried at 80 °C under vacuum to afford the material quantitatively for further characterization.

3. Characterization of PSF materials

3.1. Comparison of DRIFT IR spectra of PSFs

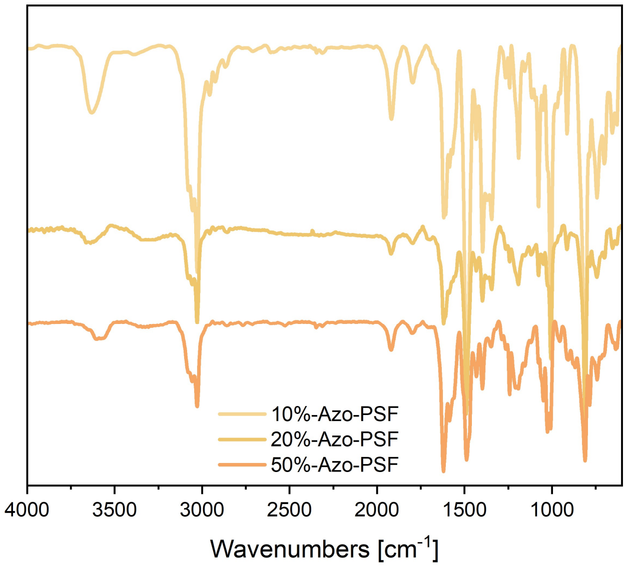

Fig. S5: DRIFT-IR spectra of **Azo-PSFs** in the region between 4000 cm^{-1} to 600 cm^{-1}.

3.2. Elemental analysis

Table S1. Elemental analysis of **Azo-PSFs**,

Sample	C (%)		H (%)		N (%)		Azo Fract %	
	Exp.	Calc.	Exp.	Calc.	Exp.	Calc.	Exp.	Calc.
10% Azo-PSF	93.35	94.24	5.80	4.99	0.85	0.77	10.2	9.1
20% Azo-PSF	93.18	93.54	5.61	4.87	1.21	1.59	14.9	20
50% Azo-PSF	91.47	91.94	4.95	4.61	3.58	3.46	52.4	50

3.3. Thermogravimetry Analysis of ^{20}Azo-PSF

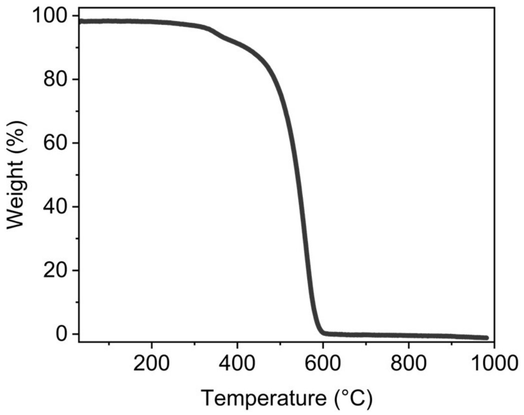

Fig. S6: Thermogravimetry of ^{20}**Azo-PSF** measured in dry air (50 mL/min) between 30°C and 1000°C.

3.4. SEM images of Azo-PSF materials

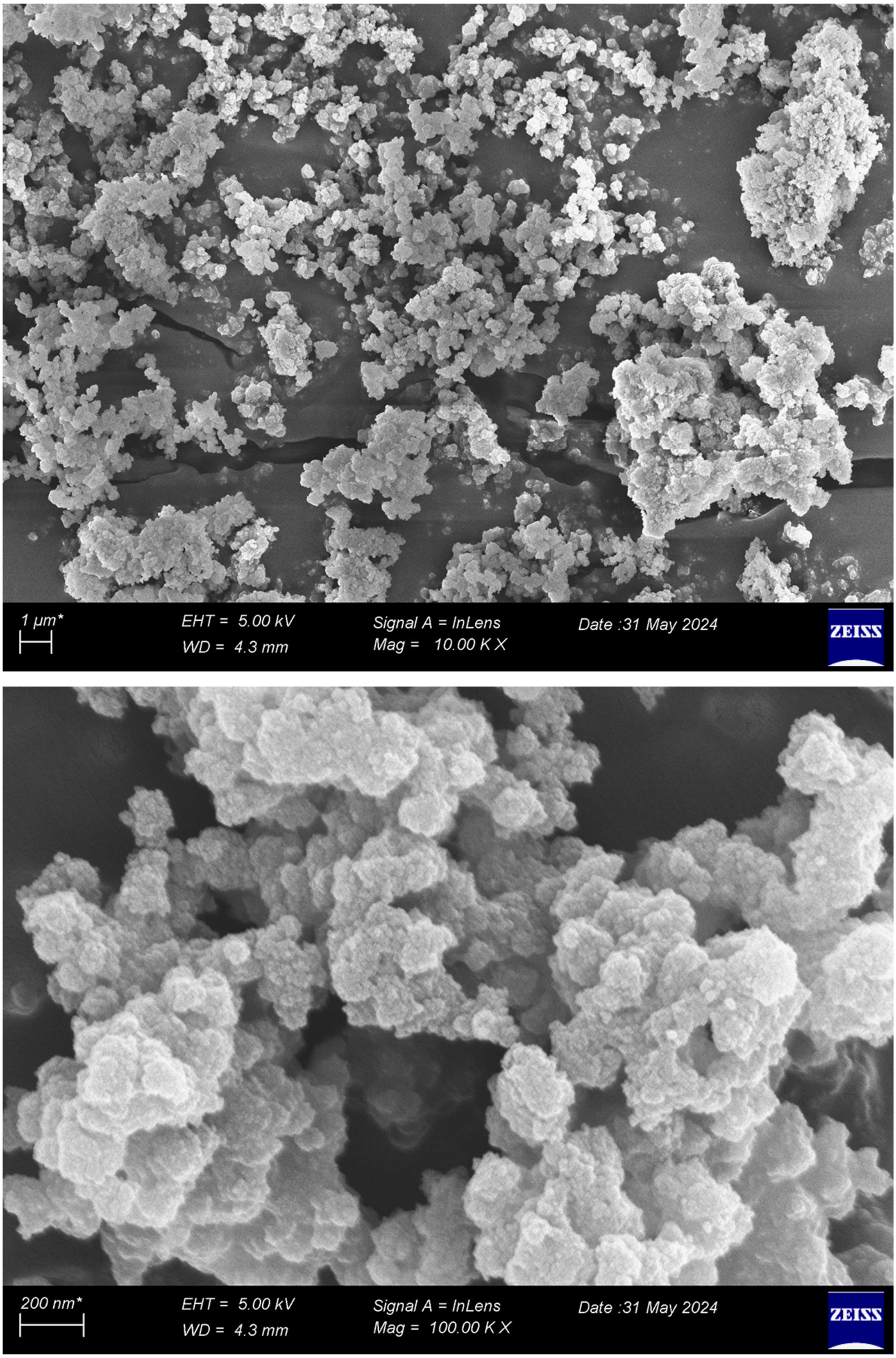

Fig. S7: SEM images of **^{10}Azo-PSF**.

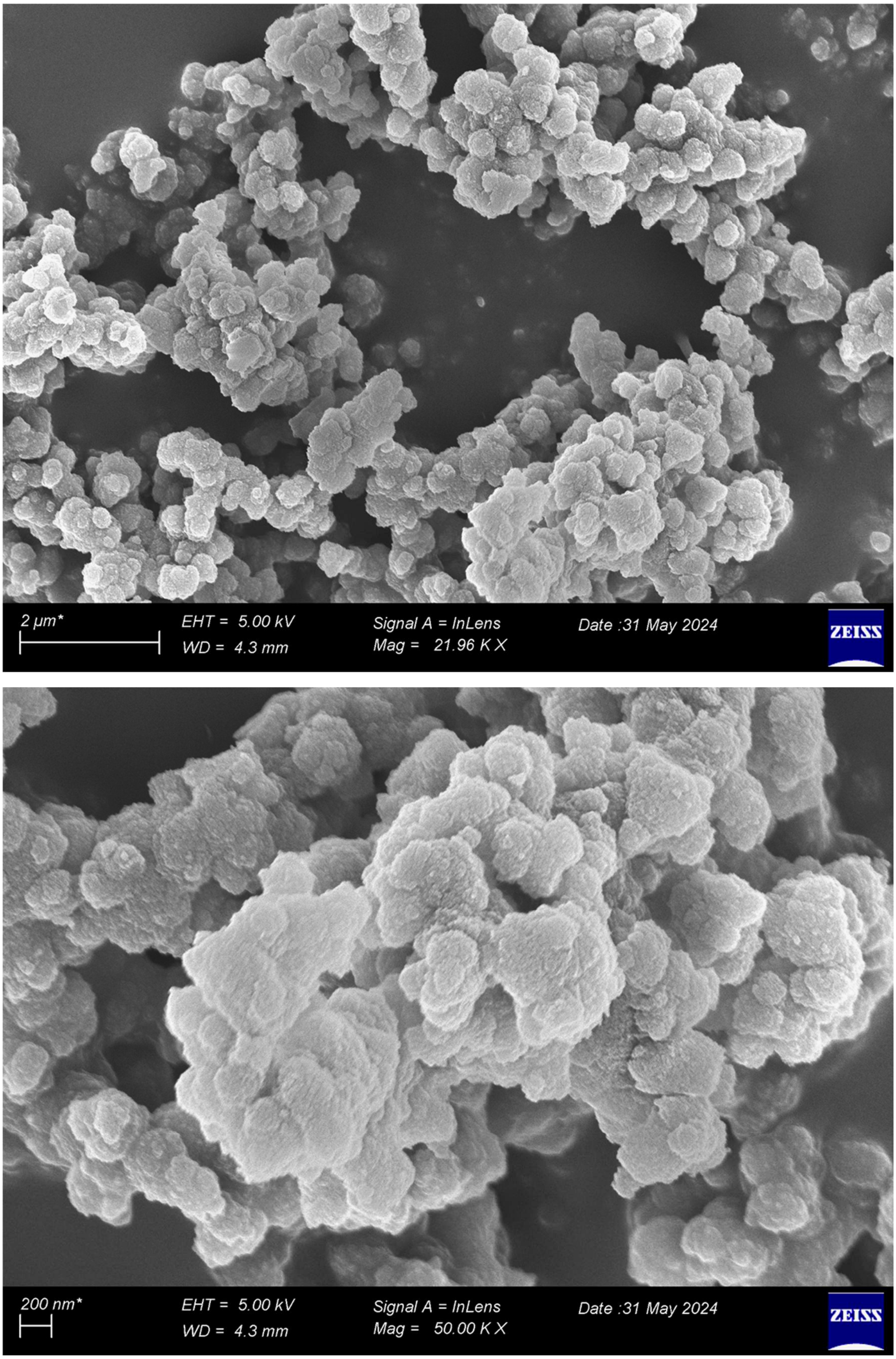

Fig. S8: SEM images of **^{20}Azo-PSF**.

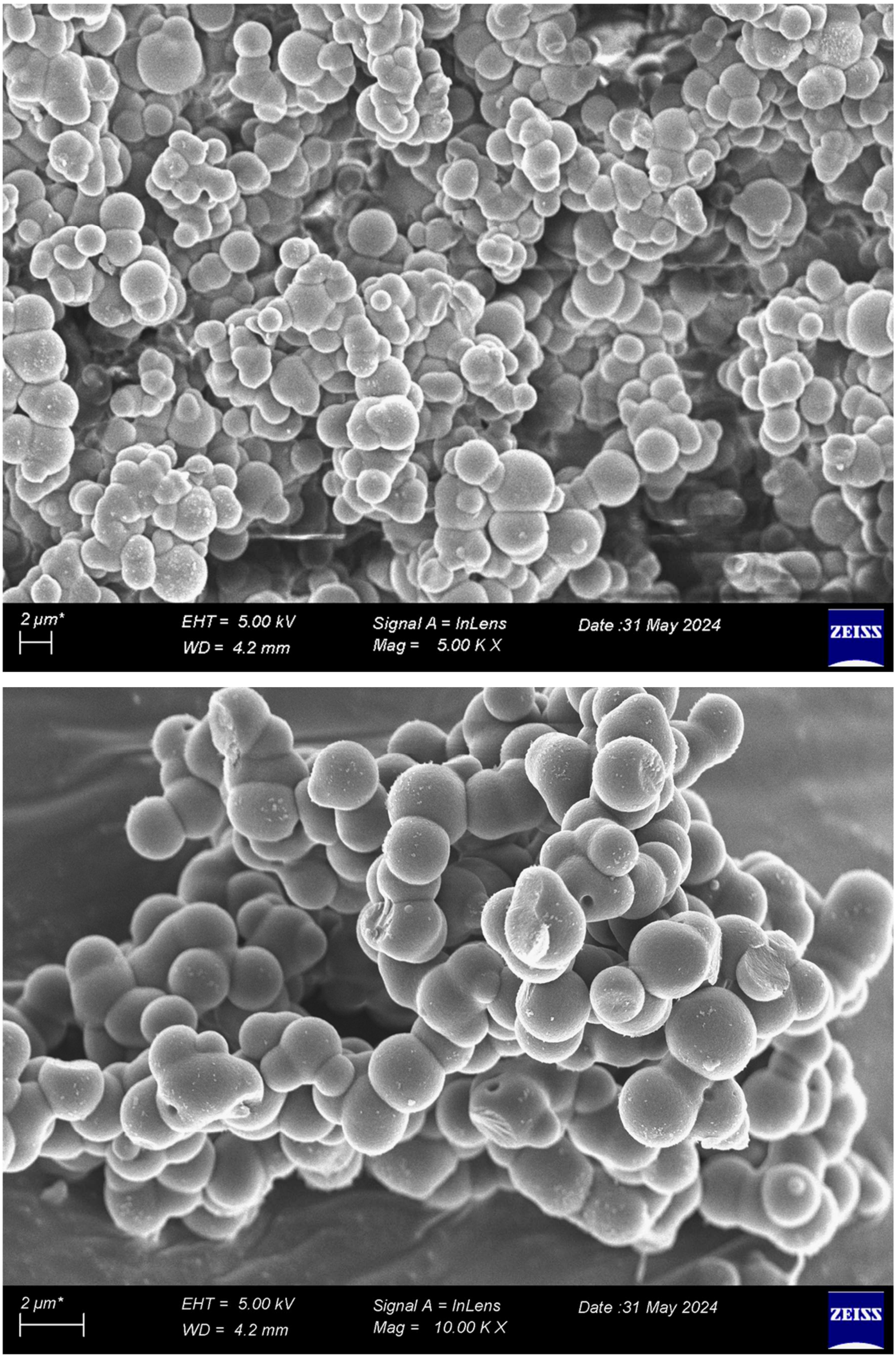

Fig. S9: SEM images of ^{50}**Azo-PSF**.

3.5. Powder X-ray diffraction (PXRD)

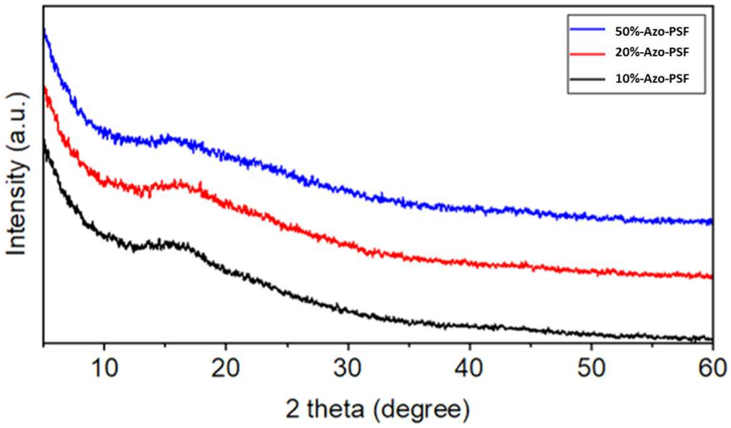

Fig S10: PXRD of **Azo-PSF** samples collected from 5 to 80 2θ degree.

3.6 Adsorption properties

Table S2: Sorption properties calculated from nitrogen adsorption isotherms collected at 77 K for the samples.

Sample	Langmuir surface area (m²/g)[1]	BET surface area (m²/g)[1]	Pore volume (cm³/g)[2]	Micropore volume (cm³/g)[3]
[10]Azo-PSF	934	845	0.35	0.30
[20]Azo-PSF	728	648	0.28	0.24
[50]Azo-PSF	42	37	-	-

[1]BET surface area was evaluated according to the Rouquerol criterion. Langmuir surface area was evaluated in the same pressure range. [2] Pore size distribution and cumulative pore volume up to 50 nm (500 Å) was calculated according to HS-2D-NLDFT theory using a carbon slit pore model as implemented in the MicroActive software. [3] Micropore volume was evaluated up to 2 nm (20 Å) according to IUPAC definition.

4. Isomerization studies

4.1. Photoisomerization in solution

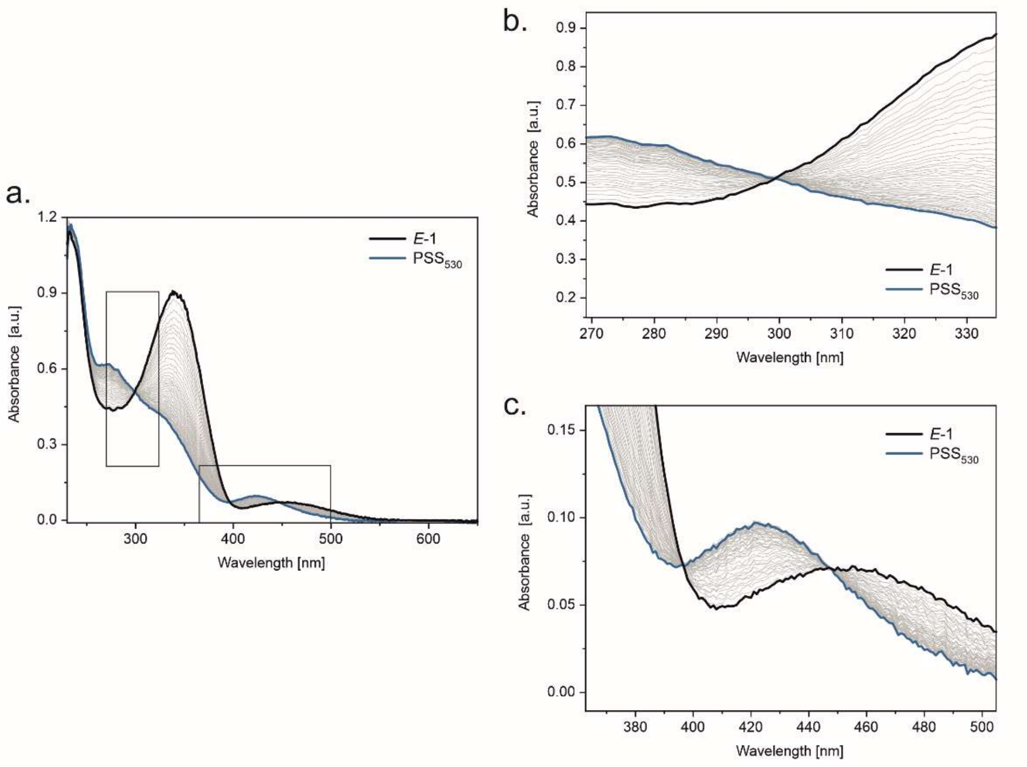

Fig. S11. a Changes in the UV/Vis absorption spectrum of ***E*-1** upon photochemical isomerisation in DCM . **c-b** Enlarged spectra showing isosbestic points.

4.2. Thermal isomerization studies of building block *E*-1.

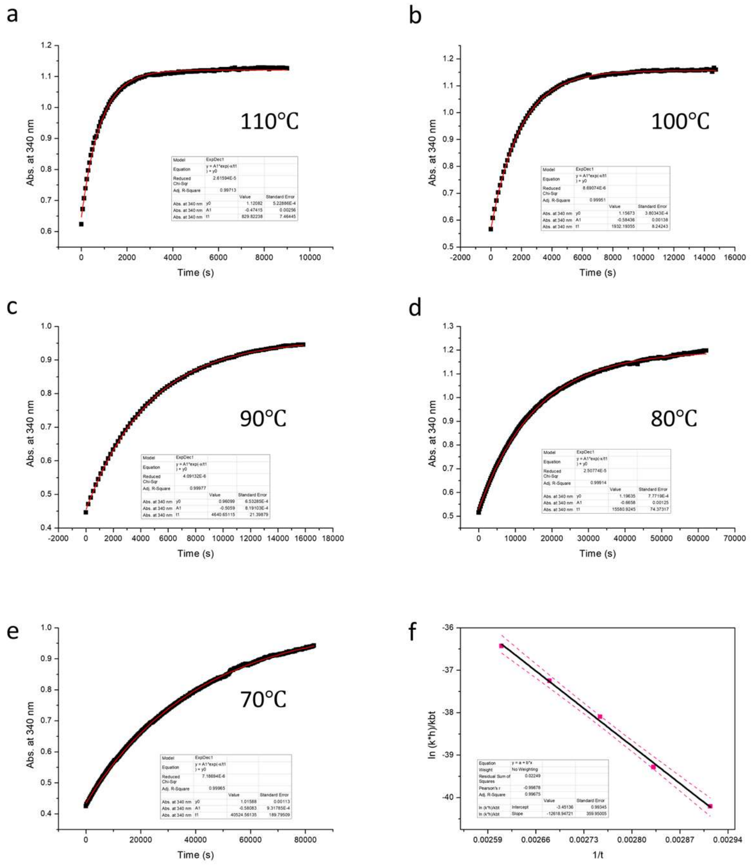

Fig. S12: **(a)** to **(e)** Thermal $Z \rightarrow E$ isomerization of azobenzene building block **1** monitored at 340 nm at five different temperatures from 110 °C to 70°C in DMSO. **f** Eyring plot analysis of thermal relaxation from *Z* isomer to *E* isomer in DMSO. The calculated thermal half-life of azobenzene linker at 298.15 K is 100 days and at 333.15 K is 24.7 hours. Dashed lines indicate 95% confidence intervals.

4.3. Reversible photoisomerization study of 50%-Azo-PSF.

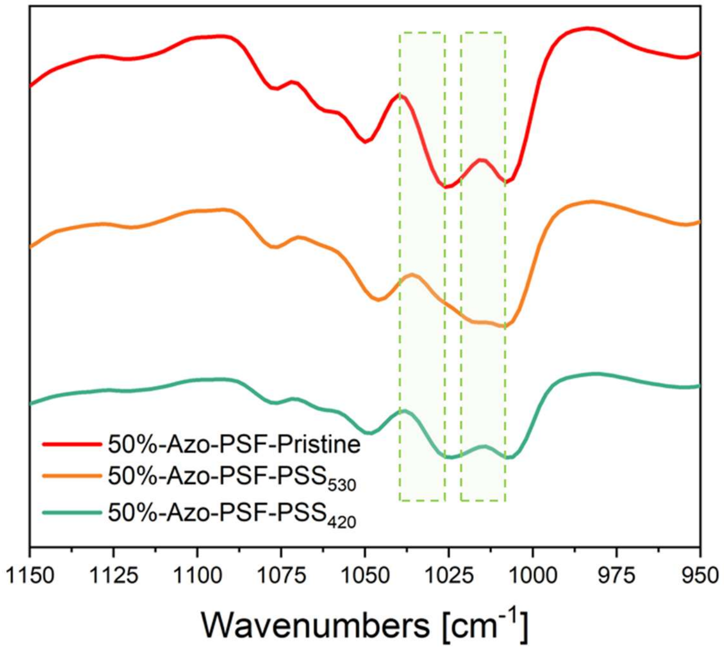

Fig. S13: DRIFT-IR spectra of ^{50}**Azo-PSF** between 1400 cm^{-1} and 1100 cm^{-1} before and after 530 nm irradiation and recovered with 420 nm light irradiation.

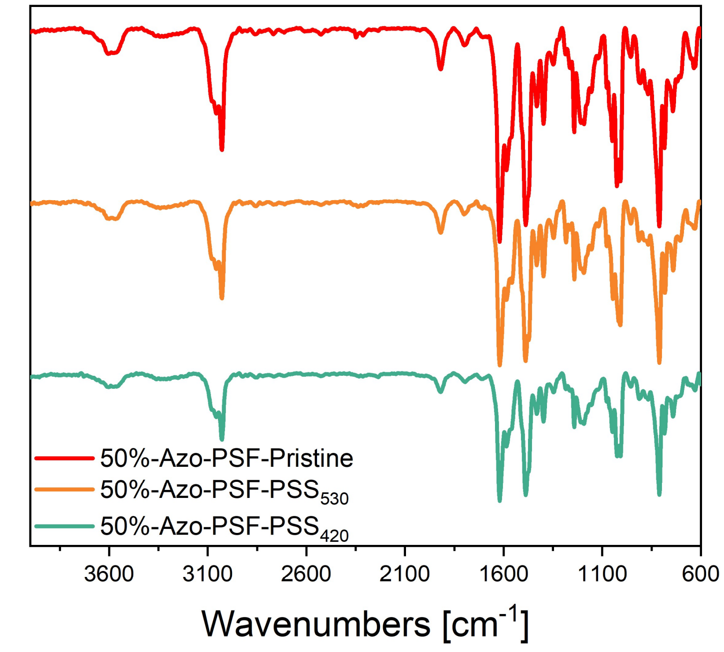

Fig. S14: DRIFT-IR spectra of ^{50}**Azo-PSF** between 4000 cm^{-1} and 600 cm^{-1} before and after 530 nm irradiation and recovered with 420 nm light irradiation.

4.4. Solid State NMR spectra

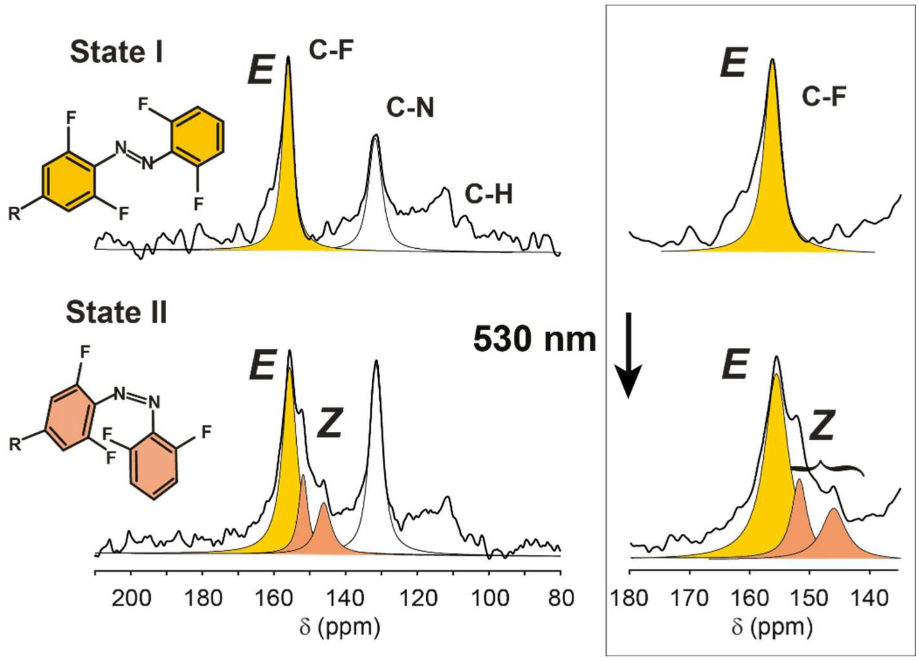

Fig. S15: Photoswitching behavior of **[20]Azo-PSF** monitored by ^{19}F-^{13}C CP MAS NMR spectra (left). Enlargement of the 135-180 ppm region to highlight *E*-to-*Z* isomerization (right). The spectra were collected at 298 K at a spinning speed of 12.5 kHz with contact time of 5 ms.

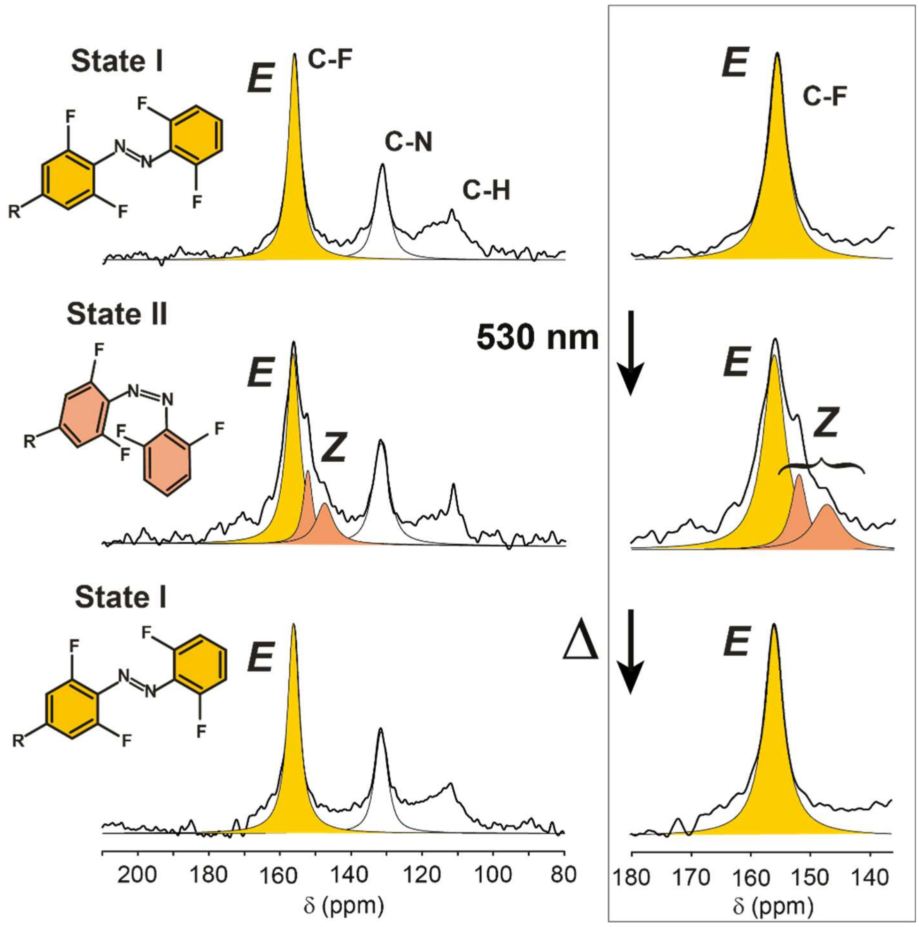

Fig. S16: Photoswitching behavior of **^{10}Azo-PSF** monitored by ^{19}F-^{13}C CP MAS NMR spectra (left). Enlargement of the 135-180 ppm region to highlight *E*-to-*Z* isomerization (right). The spectra were collected at 298 K at a spinning speed of 12.5 kHz with contact time of 5 ms.

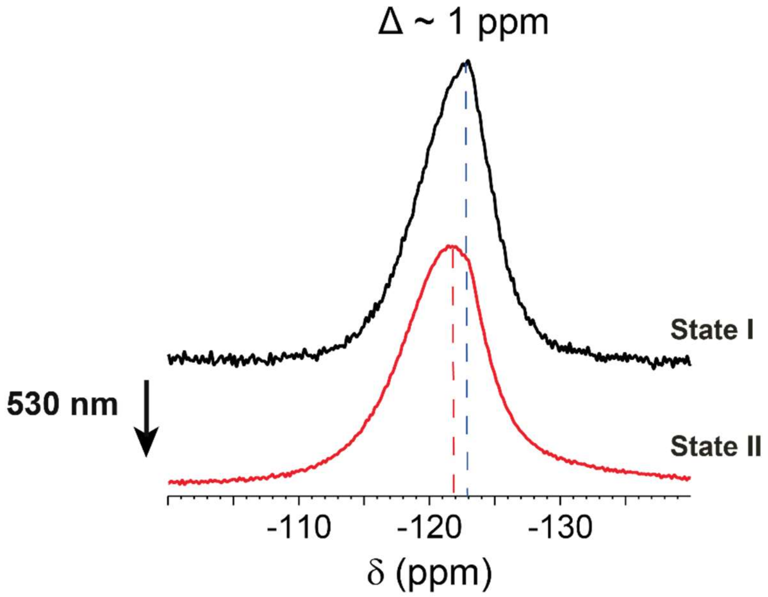

Fig. S17: Photoswitching behavior of **^{20}Azo-PSF** monitored by ^{19}F MAS NMR spectra performed at 298 K at a spinning speed of 12.5 kHz with a recycle delay of 20 s. A downfield shift of 1 ppm is observed upon irradiation at 530 nm.

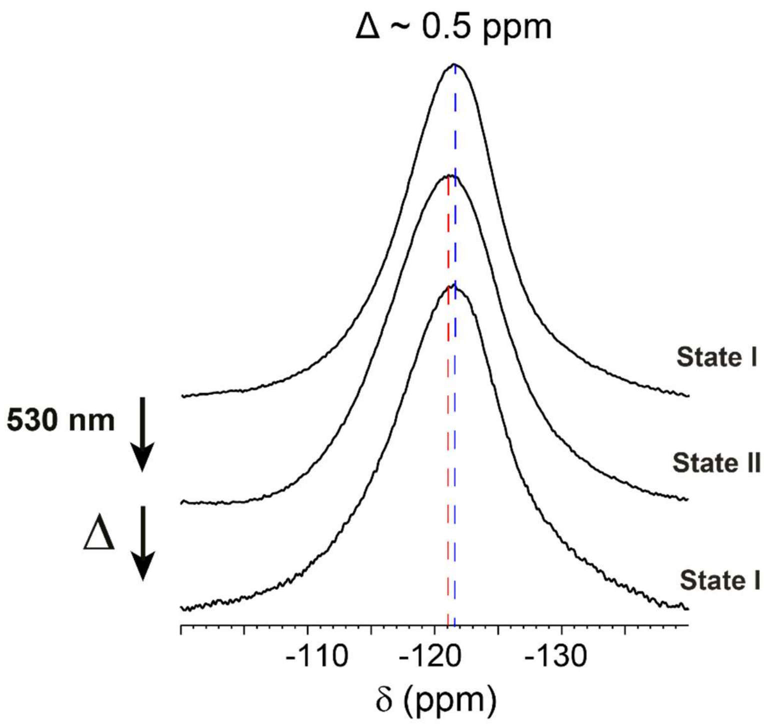

Fig. S18: Photoswitching behavior of **[50]Azo-PSF** monitored by ^{19}F MAS NMR spectra performed at 298 K at a spinning speed of 12.5 kHz with a recycle delay of 20 s. A downfield shift upon irradiation at 530 nm and the subsequent upfield shift upon thermal treatment are observed.

4.5. Gas adsorption modulation

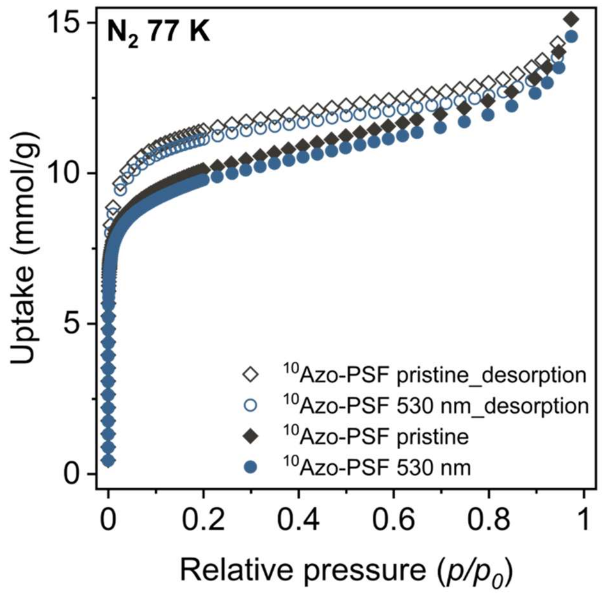

Fig. S19: N_2 adsorption isotherms measured at 77 K for pristine **[10]Azo-PSF** (grey diamonds) and after irradiation at 530 nm (blue circles).

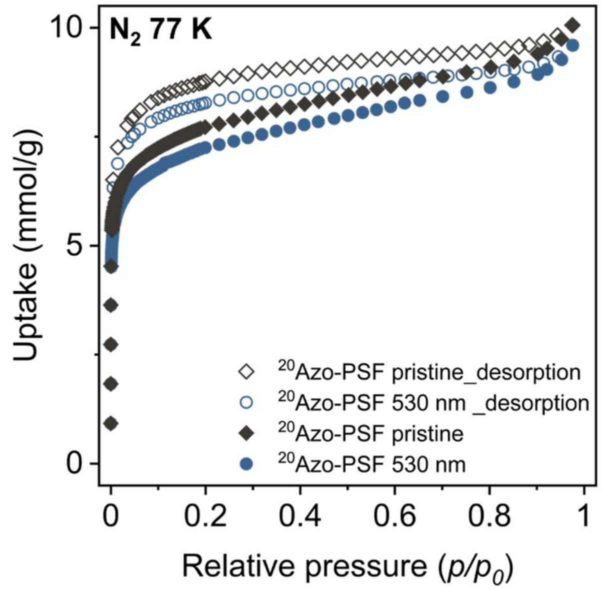

Fig. S20: N_2 adsorption isotherms measured at 77 K for pristine **[20]Azo-PSF** (grey diamonds) and after irradiation at 530 nm (blue circles).

Table S3. Textural properties of pristine **Azo-PSFs** and after irradiation at 530 nm.

Sample	Langmuir surface area (m^2/g)[1]	BET surface area (m^2/g)[1]	Pore volume (cm^3/g)[2]	Micropore volume (cm^3/g)[3]
^{10}Azo-PSF pristine	934	845	0.35	0.30
^{10}Azo-PSF 530 nm	904	818	0.34	0.29
^{20}Azo-PSF pristine	728	648	0.28	0.24
^{20}Azo-PSF 530 nm	670	607	0.26	0.22

[1]BET surface area were evaluated according to the Rouquerol criterion. Langmuir surface area were evaluated in the same pressure range. [2] Pore size distribution and cumulative pore volume up to 50 nm (500 Å) was calculated according to HS-2D-NLDFT theory using a carbon slit pore model as implemented in the MicroActive software. [3] Micropore volume was evaluated up to 2 nm (20 Å) according to IUPAC definition.

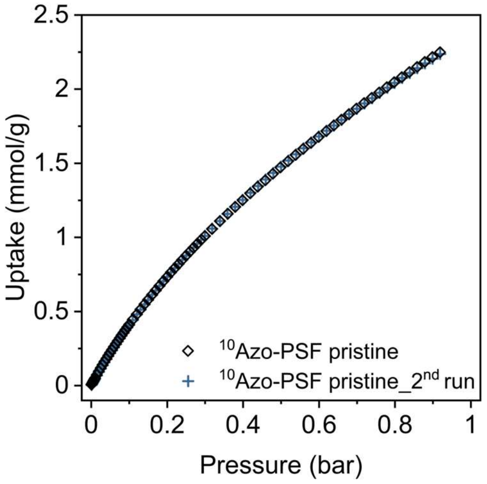

Fig. S21: CO_2 adsorption cyclability of ^{10}**Azo-PSF**. CO_2 adsorption isotherms measured at 273 K for pristine ^{10}**Azo-PSF**: first adsorption isotherm (black diamonds) and second adsorption isotherm (blue crosses). ^{10}**Azo-PSF** was activated under high vacuum (p ≤ 3μbar) overnight after the first CO_2 adsorption isotherm to remove the adsorbed species.

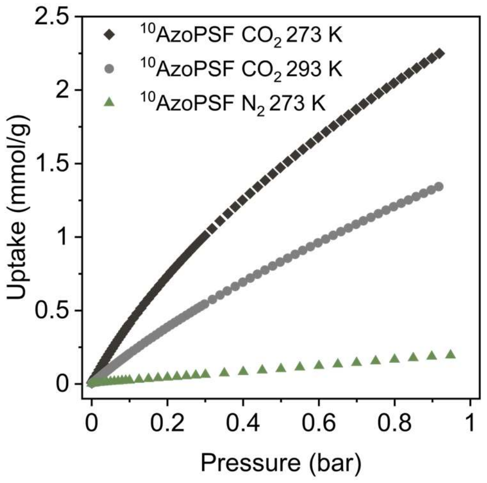

Fig. S22: CO_2 adsorption isotherms of ^{10}**Azo-PSF** measured at 273 K (black diamonds) and 293 K (light grey circles), and N_2 adsorption isotherm measured at 273 K (green triangles).

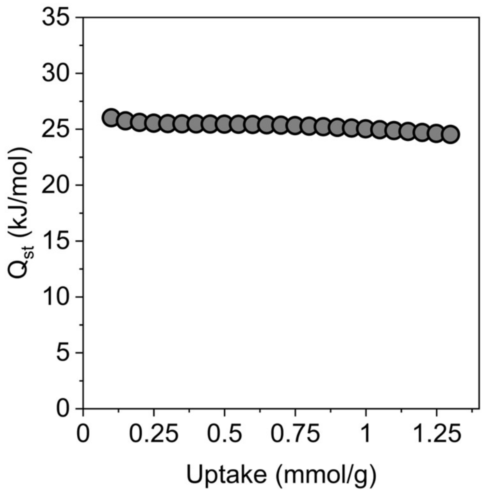

Fig. S23: CO_2 isosteric heat of adsorption of **[10]AzoPSF** calculated using the Van't Hoff equation from the CO_2 isotherms measured at 273 K and 293 K. The experimental isotherms were fitted using a dual-site Langmuir-Freundlich function.

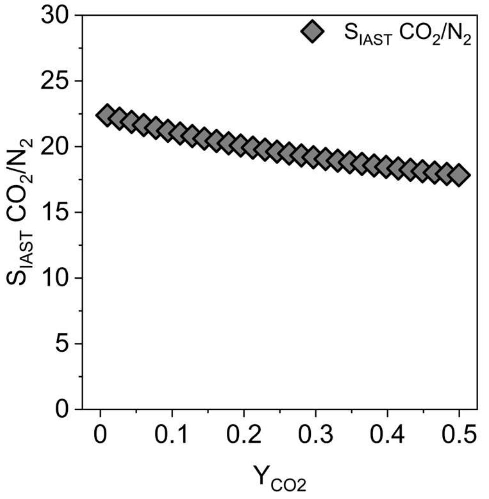

Fig. S24: CO_2/N_2 selectivity calculated according to the IAST theory from the single-component CO_2 and N_2 adsorption isotherms measured at 273 K.

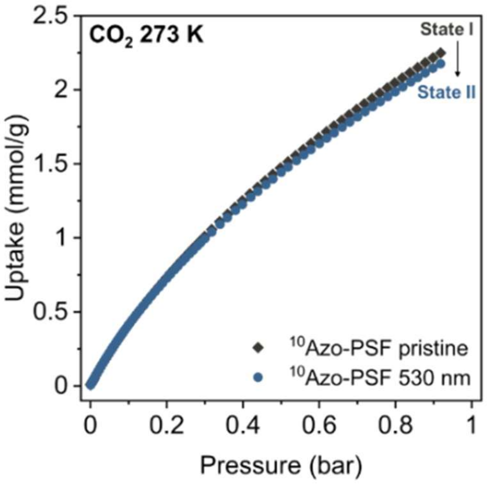

Fig. S25: CO_2 adsorption isotherms measured at 273 K for pristine **[10]Azo-PSF** (grey diamonds) and after irradiation at 530 nm (blue circles).

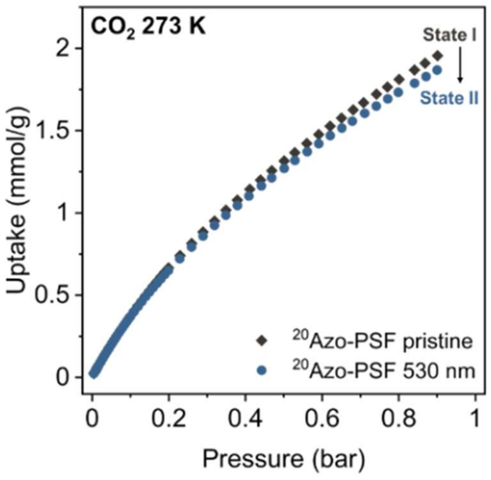

Fig. S26: CO_2 adsorption isotherms measured at 273 K for pristine **[20]Azo-PSF** (grey diamonds) and after irradiation at 530 nm (blue circles).

4.6. Photochemical isomerization of 1 under ambient light

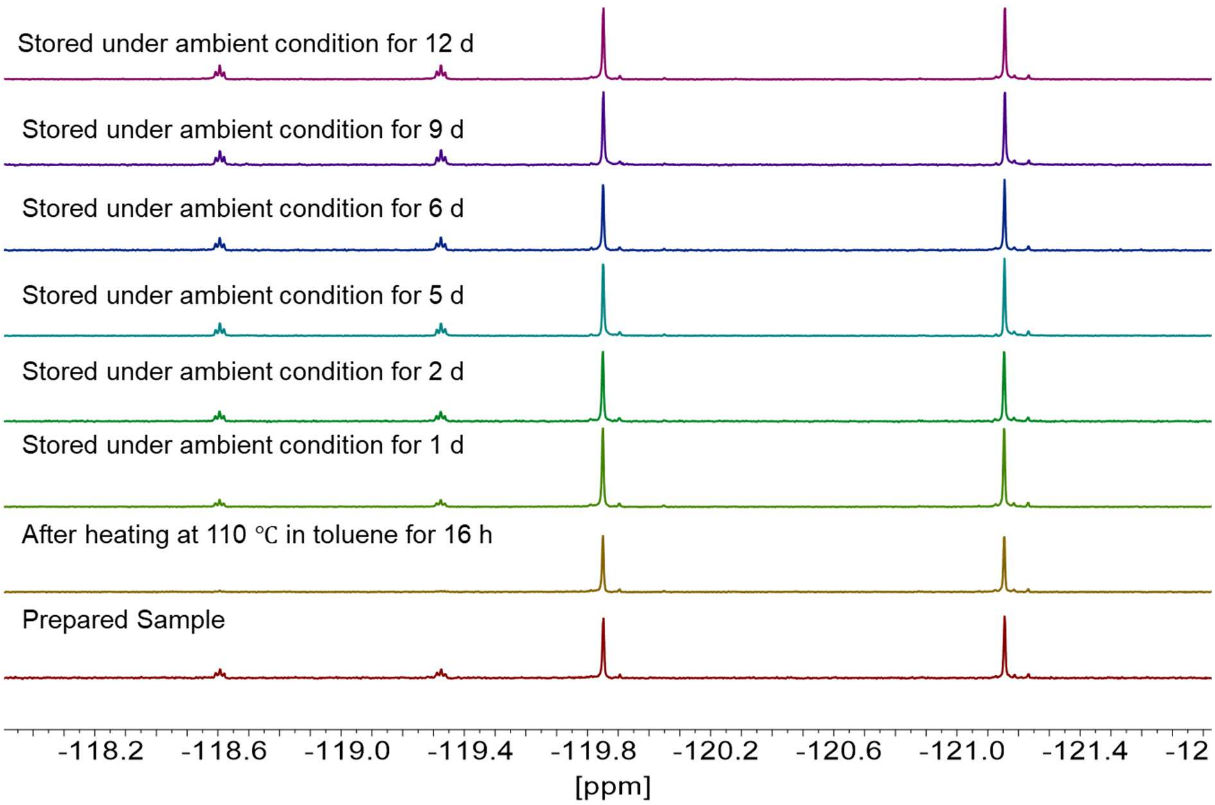

Fig. S27: ^1H NMR study for pristine compound **1** and stability study after heating at 110 ℃ in toluene for 16 h, followed by storing under ambient conditions.

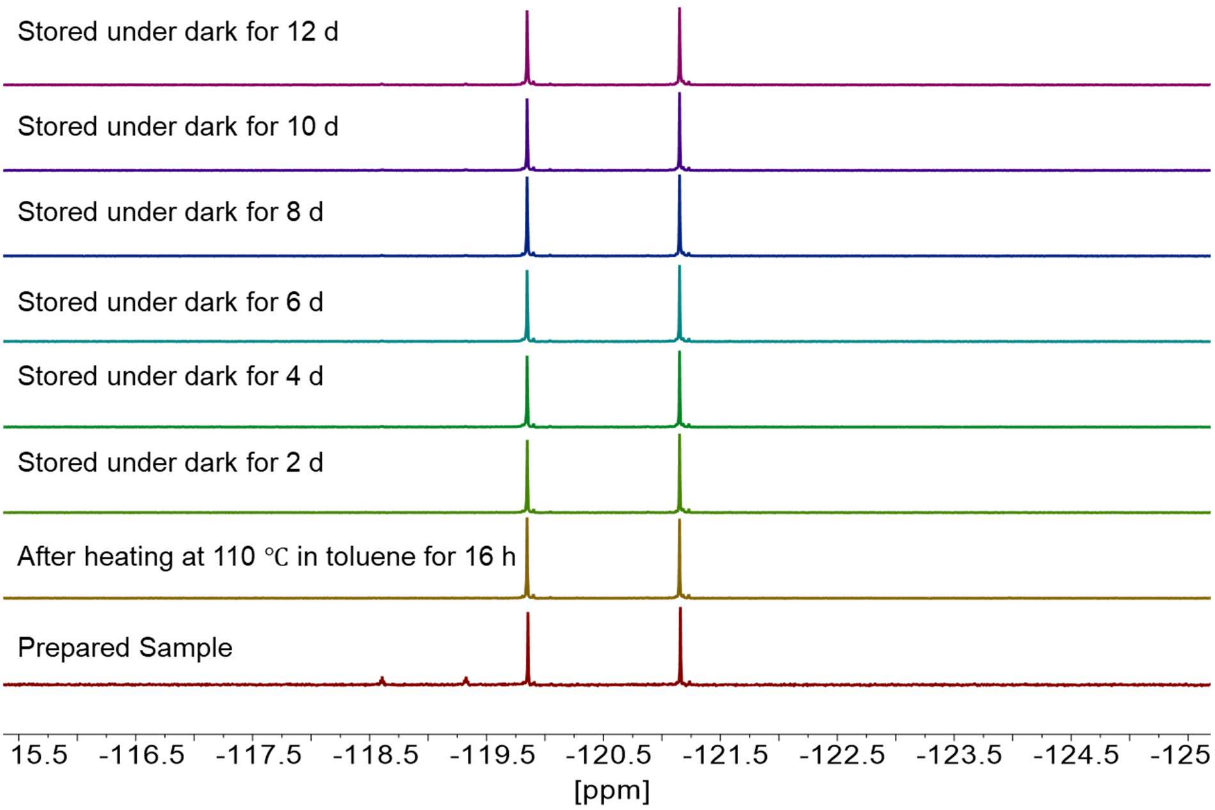

Fig. S28: ^{1}H NMR study for pristine compound **1** and stability study after heating at 110 ℃ in toluene for 16 h, followed by storing in darkness (covered fully with aluminum foil).

Table S4. Selected examples of light-responsive porous frameworks.

	Sample	Type	Photoswitch	Wave-length	Initial CO_2 uptake (mmol/g)	CO_2 uptake modulation (mmol/g)	% Amount of Modulation	Conditi-ons	Ref.
1	^{10}Azo-PSF	PAF	Tetra-*ortho*-fluoro-azobenzene	vis	2.25<br>(273 K, 0.9 bar)	-0.08<br>(273 K, 0.9 bar)	-3.5% (273 K, 0.9 bar)	*ex situ*	*This work*
2	^{20}Azo-PSF	PAF	Tetra-*ortho*-fluoro-azobenzene	vis	1.96<br>(273 K, 0.9 bar)	-0.11<br>(273 K, 0.9 bar)	-5.6% (273 K, 0.9 bar)	*ex situ*	*This work*
3	^{50}Azo-PSF	PAF	Tetra-*ortho*-fluoro-azobenzene	vis	1.5<br>(273 K, 0.9 bar)	-0.22<br>(273 K, 0.9 bar)	-14% (273 K, 0.9 bar)	*ex situ*	*This work*
4	PSF	PAF	Tetra-*ortho*-fluoro-azobenzene<br><br>spiropyran	vis	2.01 (273 K)<br>1.28 (293 K)	-0.19 (273 K)<br>-0.17 (293 K)	-9% (273 K)<br>-14% (293 K)	*ex situ*	1
5	DArE@PAF-1 (5%)	PAF	diarylethene	UV	-	-0.13 (303 K)	-16% (303 K)	*in situ*	2
6	DArE@PAF-1 (50%)	PAF	diarylethene	UV	-	-0.06 (303 K)	-26% (303 K)	*in situ*	2
7	PCTF$_2$	COF	Azobenzene	UV	2.17<br>(273 K, 1.1 bar)	-0.38 (273 K, 1.1 bar)	-18% (273 K, 1.1 bar)	*ex situ*	3
8	mPCTF$_1$	COF	Azobenzene	UV	2.19 (273 K, 1.1 bar)	-0.32 (273 K, 1.1 bar)	-15% (273 K, 1.1 bar)	*ex situ*	3
9	PCTF$_1$	COF	Azobenzene	UV	1.49<br>(273 K, 1.1 bar)	-0.15 (273 K, 1.1 bar)	-10% (273 K, 1.1 bar)	*ex situ*	3
10	[4F-Azo]X-TPB-DMTP-	COF	Tetra-*ortho*-fluoro-azobenzene	vis	4.22 (273 K)	+1.0 (273 K)	+24% (273 K)	*ex situ*	4

	COFs (x = 0.5)								
11	[4F-Azo]X-TPB-DMTP-COFs (x = 0.67)	COF	Tetra-*ortho*-fluoro-azobenzene	vis	3.22 (273 K)	+0.83 (273 K)	+ 27% (273 K)	*ex situ*	4
12	PCN-123	MOF	Azobenzene	UV	1.02 (295 K)	-0.55 (295 K)	-54% (295 K)	*ex situ*	5
13	Zn(AzDC)(4,4'-BPE)$_{0.5}$	MOF	Azobenzene	UV	-	-	-42%	*in situ*	6
14	ECUT-15	MOF	Azobenzene ECUT-15	UV	0.28 (298 K)	-0.14 (298 K)	-45% (298 K)	*in situ*	7
15	F-azo-UiO-66(Zr	MOF	Tetra-*ortho*-fluoro-azobenzene	vis	-	-	-	*in situ*	8
16	F-azo-MIL-53(Al	MOF	Tetra-*ortho*-fluoro-azobenzene	vis	0.85 (273 K, 1.2 bar, λ>500 nm)	-0.085 (273 K, 1.2 bar, λ>500 nm)<br><br>-0.14 (273 K, 1.2 bar, λ>300 nm)	-10% (273 K, 1.2 bar, λ>500 nm)<br><br>-15% (273 K, 1.2 bar, λ>300 nm)	*in situ*	8
17	MOF-808-SP	MOF	Spiropyran	UV	1.34 (298 K, 1.2 bar)	+ 0.23 (298 K, 1.2 bar)	+ 17% (298 K, 1.2 bar)	*ex situ*	9
18	Fe_2CoPCN-250	MOF	Azobenzene	UV	2.92 (298 K, 850 mbar)	- 1.60 (298 K, 850 mbar)	-56% (298 K, 850 mbar)	*in situ*	10
19	[Zn_2(3,3'-bpeab)(oba)$_2$]·DMF	MOF	Azobenzene	UV	1.69 (298 K, 1.2 bar)	- 0.26 (298 K, 1.2 bar)	-37% (298 K, 1.2 bar)	*in situ*	11
20	Magnetic PCN-250	MOF	Azobenzene	UV	-	-	-29.4/-30.7% (298 K) depending on the amount of magnetic NPs	*in situ*	12
21	ECUT-30a	MOF	Azobenzene<br><br>diarylethene	UV	0.87 (293 K)	-0.25 (293 K)	-28.6% (293 K)	*in situ*	13
22	^{Azo}MOF	MOF	Azobenzene	UV	19.5 (195 K)	-4.1 (195 K)	- ~20% (195 K)	*ex situ*	14
23	[Zn_2(bdc)$_2$(DTE)]	MOF	Dithienylethene	UV	6.25 (195 K)	-2.28 (195 K)	-36% (195 K)	*ex situ*	15
24	DMOF3	MOF	Dithienylethene	UV	-	-	- ~50% (298 K, 20 bar)	*ex situ*	16
25	JUC-62	MOF	Azobenzene	UV	2.05 (298 K)	-0.71 (298 K)	-34% (298 K)	*in situ*	17
26	Azo-UiO-66	MOF	Azobenzene	UV	0.76 (298 K)	-0.2 (298 K)	-33% (298 K)	*in situ*	18
27	Azo-DMOF-1	MOF	Azobenzene	UV	1.83 (298 K)	-0.54 (298 K)	-30% (298 K)	*in situ*	19
28	T(7.5)/U-azo	MOF	Azobenzene	UV	1.94 (273 K)	-0.61 (273 K)	-45.6% (273 K)	-	20
29	Azo(33.3)	MOF	Azobenzene	UV	1.87 (298 K)	-0.47 (298 K)	-27% (298 K)	-	21
30	Azo(66.7)	MOF	Azobenzene	UV	0.89 (298 K)	-0.29 (298 K)	-33.7% (298 K)	-	21
31	PCN-250-Al	MOF	Azobenzene	UV	4.73 (298 K)	-2.65 (298 K)	-56.1% (273 K)	*in situ*	22
32	PCN-250-In	MOF	Azobenzene	UV	3.08 (298 K)	-1.10 (298 K)	-35.8% (273 K)	*in situ*	22
33	PCN-250-Sc	MOF	Azobenzene	UV	3.73 (298 K)	-1.16 (298 K)	-31.1% (273 K)	*in situ*	22
34	PCN-250-Fe(II/III)	MOF	Azobenzene	UV	3.31 (298 K)	-1.36 (298 K)	-41.1% 273 K	*in situ*	22
35	PCN-250-Fe(III)	MOF	Azobenzene	UV	1.65 (298 K)	-0.25 (298 K)	-15.1% (273 K)	*in situ*	22
36	U-mazo	MOF	Azobenzene	UV	0.83 (273 K)<br>-43% (273 K)	-0.34 (273 K)	-43% (273 K)	*ex situ*	23
37	NUT-102	MOC	Azobenzene	UV	1.47 (273 K)	-0.58 (273 K)	-65% (273K)	*in situ*	24

References to Table S5:

[1] J. Sheng, J. Perego, S. Bracco, P. Cieciórski, W. Danowski, A. Comotti, B. L. Feringa, Orthogonal Photoswitching in a Porous Organic Framework. *Angew. Chem. Int. Ed.* **2024**, *63*, e202404878.

[2] R. Lyndon, K. Konstas, R. A. Evans, D. J. Keddie, M. R. Hill, B. P. Ladewig, Tunable Photodynamic Switching of DArE@PAF-1 for Carbon Capture. *Adv. Funct. Mater*. **2015**, *25*, 4405-4411.
[3] Q. Huang, Z. Zhan, R. Sun, J. Mu, B. Tan, C. Wu, Light Triggered Pore Size Tuning in Photoswitching Covalent Triazine Frameworks for Low Energy CO_2 Capture. *Angew. Chem. Int. Ed*. **2023**, 62, e202305500.
[4] Y. Feng, G. Wang, R. Liu, X. Ye, S. Tao, M. A. Addicoat, Z. Li, Q. Jiang, D. Jiang, Photoresponsive Covalent Organic Frameworks: Visible-Light Controlled Conversion of Porous Structures and Its Impacts. *Angew. Chem. Int. Ed*. **2024**, *63*, e202400009.
[5] J. Park, D. Yuan, K. T. Pham, J.-R. Li, A. Yakovenko, H.-C. Zhou, Reversible Alteration of CO2 Adsorption upon Photochemical or Thermal Treatment in a Metal–Organic Framework. *J. Am. Chem.Soc.* **2012**, *134*, 99−102.
[6] R. Lyndon, K. Konstas, B. P. Ladewig, P. D. Southon, C. J. Kepert, M. R. Hill, Dynamic Photo-Switching in Metal–Organic Frameworks as a Route to Low-Energy Carbon Dioxide Capture and Release. *Angew. Chem. Int. Ed.* **2013**, *52*, 3695 –3698.
[7] L. L. Gong, X. F. Feng, F. Luo, Novel azo-Metal–Organic Framework Showing a 10-Connected bct Net, Breathing Behavior, and Unique Photoswitching Behavior toward CO_2. *Inorg. Chem.* **2015**, 54, 24, 11587−11589.
[8] S. Castellanos, A. Goulet-Hanssens, F. Zhao, A. Dikhtiarenko, A. Pustovarenko, S. Hecht, J. Gascon, F. Kapteijn, D. BlØger, Structural Effects in Visible-Light-Responsive Metal–Organic Frameworks Incorporating ortho-Fluoroazobenzenes. *Chem. Eur. J.* **2016**, *22*, 746-752.
[9] K. Healey, W. Liang, P. D. Southon, T. L. Church, D. M. D'Alessandro, Photoresponsive spiropyran-functionalised MOF-808: postsynthetic incorporation and light dependent gas adsorption properties. *J. Mater. Chem. A* **2016**, *4*, 10816–10819
[10] H. Li, M. R. Martinez, Z. Perry, H.-C. Zhou, P. Falcaro, C. Doblin, S. Lim, A. J. Hill, B. Halstead, M. R. Hill, A Robust Metal–Organic Framework for Dynamic Light-Induced Swing Adsorption of Carbon Dioxide. *Chem. Eur. J.* **2016**, *22*, 11176-11179.
[11] W.-C. Song, X.-Z. Cui, Z.-Y. Liu, E.-C. Yang, X.-J. Zhao, Light-triggered Supramolecular Isomerism in a Self-catenated Zn(II)-organic Framework: Dynamic Photo-switching CO_2 Uptake and Detection of Nitroaromatics. *Sci. Rep.* **2016**, *6*, 34870.
[12] H. Li, M. M. Sadiq, K. Suzuki, C. Doblin, S. Lim, P. Falcaro, A. J. Hill, M. R. Hill, MaLISA – a cooperative method to release adsorbed gases from metal–organic frameworks. *J. Mater. Chem. A* **2016**, *4*, 18757-18762.
[13] C. B. Fan, Z. Q. Liu, L. L. Gong, A. M. Zheng, L. Zhang, C. S. Yan, H. Q. Wu, X. F. Feng, F. Luo, Photoswitching adsorption selectivity in a diarylethene–azobenzene MOF. *Chem. Commun.*, **2017**, *53*, 763-766.
[14] H. Huang, H. Sato, T. Aida, Crystalline Nanochannels with Pendant Azobenzene Groups: Steric or Polar Effects on Gas Adsorption and Diffusion? *J. Am. Chem. Soc.,* **2017**, 139, 26, 8784−8787.
[15] Y. Zheng, H. Sato, P. Wu, H. J. Jeon, R. Matsuda, S. Kitagawa, Flexible interlocked porous frameworks allow quantitative photoisomerization in a crystalline solid. *Nature Commun.* **2017**, *8*, 100.
[16] V. I. Nikolayenko, S. A. Herbert, L. J. Barbour, Reversible structural switching of a metal–organic framework by photoirradiation. *Chem. Commun.* **2017**, *53*, 11142—11145.
[17] N. Prasetya, B. P. Ladewig, Dynamic photo-switching in light-responsive JUC-62 for CO_2 capture. *Sci. Rep*. **2017**, *7*, 13355.
[18] N. Prasetya, B. C. Donose, B. P. Ladewig, A new and highly robust light-responsive Azo-UiO-66 for highly selective and low energy post-combustion CO_2 capture and its application in a mixed matrix membrane for CO_2/N_2 separation. *J. Mater. Chem. A*, **2018**, *6*, 16390–16402.
[19] N. Prasetya, B. P. Ladewig, New Azo-DMOF-1 MOF as a Photoresponsive Low-Energy CO_2 Adsorbent and Its Exceptional CO_2/N_2 Separation Performance in Mixed Matrix Membranes. *ACS Appl. Mater. Interfaces,* **2018**, 10, *40*, 34291–34301.
[20] Y. Jiang, P. Tan, S.-C. Qi, X.-Q. Liu, J.-H. Yan, F. Fan, L.-B. Sun, Metal–Organic Frameworks with Target-Specific Active Sites Switched by Photoresponsive Motifs: Efficient Adsorbents for Tailorable CO_2 Capture. *Angew. Chem. Int. E*d**. 2019**, *58*, 6600 –6604.
[21] N. Prasetya, B. P. Ladewig, An insight into the effect of azobenzene functionalities studied in UiO-66 frameworks for low energy CO_2 capture and CO_2/N_2 membrane separation. *J. Mater. Chem. A* **2019**, *7*, 15164–15172.
[22] H. F. Drake, Z. Xiao, G. S. Day, S. W. Vali, L. L. Daemen, Y. Cheng, P. Cai, J. E. Kuszynski, H. Lin, H.-C. Zhou, M. R. Ryde, Influence of Metal Identity on Light-Induced Switchable Adsorption in Azobenzene-Based Metal–Organic Frameworks. *ACS Appl. Mater. Inter.* **2022**, *14*, 9, 11192−11199.
[23] Q. Huang, J. Mu, Z. Zhan, F. Wang, S. Jin, B. Tan, C. Wu, A steric hindrance alleviation strategy to enhance the photo-switching efficiency of azobenzene functionalized metal–organic frameworks toward tailorable carbon dioxide capture. *J. Mater. Chem. A* **2022**, *10*, 8303-8308.
[24] Y. Jiang, T. Yang, X.-Q. Liu, P. Cuia, L.-B. Sun, A metal–organic cage with light-switchable motifs for controllable CO_2 adsorption. *J. Mater. Chem. A* **2024**, *12*, 892-898.

5. References

[1] J. A. Hutchison, H. Uji-i, A. Deres, T. Vosch, S. Rocha, S. Müller, A. A. Bastian, J. Enderlein, H. Nourouzi, C. Li, A. Herrmann, K. Müllen, F. De Schryver, J. Hofkens, A surface-bound molecule that undergoes optically biased Brownian rotation. *Nat. Nanotechnol.* **2014**, *9*, 131.

[2] L. Zhang, J. Sun, F. Sun, P. Chen, J. Liu, G. Zhu, Facile Synthesis of Ultrastable Porous Aromatic Frameworks by Suzuki–Miyaura Coupling Reaction for Adsorption Removal of Organic Dyes. *Chem. Eur. J.* **2019**, *25*, 3903.

[3] D. Mutruc, A. Goulet-Hanssens, S. Fairman, S. Wahl, A. Zimathies, C. Knie, S. Hecht, Modulating Guest Uptake in Core–Shell MOFs with Visible Light. *Angew. Chem. Int. Ed.* **2019**, *58*, 12862.
